# Honokiol Dimers and Magnolol Derivatives with New Carbon Skeletons from the Roots of *Magnolia officinalis* and Their Inhibitory Effects on Superoxide Anion Generation and Elastase Release

**DOI:** 10.1371/journal.pone.0059502

**Published:** 2013-05-07

**Authors:** Hung-Cheng Shih, Tsong-Long Hwang, Hung-Chung Chen, Ping-Chung Kuo, E-Jian Lee, Kuo-Hsiung Lee, Tian-Shung Wu

**Affiliations:** 1 Department of Chemistry, National Cheng Kung University, Tainan, Taiwan; 2 Graduate Institute of Natural Products and Chinese Herbal Medicine Research Team, Healthy Aging Research Center, College of Medicine, Chang Gung University, Taoyuan, Taiwan; 3 Department of Biotechnology, National Formosa University, Yunlin, Taiwan; 4 Departments of Surgery and Anesthesiology, and Institute of Biomedical Engineering, National Cheng Kung University, Medical Center and Medical School, Tainan, Taiwan; 5 Natural Products Research Laboratories, UNC Eshelman School of Pharmacy, University of North Carolina, Chapel Hill, North Carolina, United States of America; 6 Chinese Medicinal Research and Development Center, China Medical University and Hospital, Taichung, Taiwan; 7 Department of Pharmacy, China Medical University, Taichung, Taiwan; National Research Council of Italy, Italy

## Abstract

Two honokiol dimers, houpulins A and B (**1** and **2**), and two magnolol derivatives, houpulins C and D (**3** and **4**), were isolated and characterized from an ethanol extract obtained from the roots of *Magnolia officinalis*. The chemical structures were determined based on spectroscopic and physicochemical analyses, which included 1D and 2D NMR, as well as mass spectrometry data. These four oligomers possess new carbon skeletons postulated to be biosynthesized from the coupling of three or four C_6_-C_3_ subunits. In addition, the new oligomers were evaluated for inhibition of superoxide anion generation and elastase release, and houpulin B (**2**) was identified as a new anti-inflammatory lead compound.

## Introduction

*Magnolia officinalis* Rehd. et Wils. (Magnoliaceae) is called Hou-pu in Chinese, and is a rare and endangered species listed under Class II National Protection in China. It's roots, stems, and branches are used in traditional Chinese medicine for the treatment of various disorders, such as depression coughing, asthma, liver disease, shoulder pain, urinary problems, and diarrhea [1], [2]. Neolignans, sesquiterpenes, sesquiterpene neolignans [3], [4], [5], phenylpropanoids[6], [7] have been identified from prior phytochemical studies of *M. officinalis* [4]. These constituents exhibit antimicrobial [8], [9], anticancer [10], [11], [12], anti-epileptic [13], antitumor [3], antibacterial [14], cytotoxic [6], and anti-inflammatory effects [7], [15], [16]. The various oligomeric neolignans in the plant are linked through the aromatic rings, including *ortho*, *ortho* (*o*,*o*)-linked dimers, (*o*,*o*)-linked trimers, dimers and trimers with *o*,O-linkages, and *o*,*o*-/*o*,*p*-linked trimers [17], [18], [19]. These neolignans have significant potential as new anti-inflammatory lead drugs. However, the chemical and biological studies of the *Magnolia* species have focused mainly on the constituents obtained from the stems and bark, because of the rareness of the roots. In the present work, we investigated the ethanol extract obtained from the roots of *M. officinalis* and reported two honokiol dimers and two magnolol derivatives, as well as possible biogenetic pathways. In addition, these isolates were examined for inhibition of superoxide anion generation and elastase release to evaluate their anti-inflammatory potential.

## Materials and Methods

### Ethics statement

Blood was taken from healthy human donors (20–30 years old) by venipuncture, using a protocol approved by the Institutional Review Board at Chang Gung Memorial Hospital. All donors gave written consent. The Medical Ethics Committee of Chang Gung Memorial Hospital approved this consent procedure.

### General experimental procedures

Optical rotations were measured using a JASCO DIP-370 digital polarimeter. IR spectra were obtained with a Shimadzu FT-IR DR-8011 spectrophotometer. ^1^HNMR, ^13^C NMR, COSY, HMQC, HSQC, HMBC and NOESY spectra were recorded on Bruker AVANCE III-400 spectrometer. Chemical shifts are shown in δ values (ppm) with tetramethylsilane as an internal standard. Mass spectra were measured on Bruker APEXII spectrometer with ESI ionization (positive-ion mode). Column chromatography was performed in silica gel (70–230 mesh, 230–400 mesh), and PTLC was executed on Merck pre-coated Si gel 60 F_254_ plates, using UV light to visualize the spots.

### Plant material

The root of *M. officinalis* was provided by Chuang Song-Zong Pharmaceutical Factory and authenticated by Prof. C. S. Kuo, Department of Life Science, National Cheng Kung University. A voucher specimen (2010000013) has been deposited in the Herbarium of National Cheng Kung University, Tainan, Taiwan.

### Extraction and isolation

Dried and powdered roots of *M. officinalis* (5.0 kg) were refluxed with ethanol (6×20 L) and filtered. The filtrate was concentrated to afford the crude extract (2.0 kg). The crude extract was suspended in water and partitioned with dichloromethane. The organic layers were combined and concentrated to yield a dichloromethane extract (550.0 g). The dichloromethane extract was further partitioned with 5% HCl aqueous solution to afford dichloromethane solubles (440.0 g) and 5% HCl aqueous extract (90.0 g). The dichloromethane solubles were subjected to open column chromatography over silica gel by eluting with a mixture of *n*-hexane and EtOAc (19∶1) and stepwise gradient of EtOAc to obtain 10 fractions. Fraction 6 was further purified by column chromatography over silica gel with a mixed eluent of *n*-hexane and acetone (4∶1) followed by repeated column and thin-layer chromatography to afford compounds **1** (17.4 mg) and **2** (14.7 mg). Similarly, repeated column chromatography, preparative thin-layer chromatography, and HPLC of fraction 7 yielded **3** (2.2 mg) and **4** (5.8 mg).

**Houpulin A (1):** brown syrup; [α]_D_^25^ −7.88 (c 0.83, MeOH); IR (KBr) λ_max_ 3502, 3390, 3078, 1639, 1604, 1504, 1465, 1226, 914, 756 cm^−1^; ^1^H NMR: see Table 1; ^13^C NMR: see Table 1; ESI-MS m/z: 553 ([M+Na]^+^); HR-ESI-MS m/z: 553.2357 ([M+Na]^+^) (Calcd for C_36_H_34_O_4_Na, 553.2355).

**Table 1:** ^1^H and ^13^C Spectroscopic Data of **1**–**4**.

	1^a^		2^a^		3 b		4 a	
position	δ_H_ mult. (*J*, Hz)	δ_C_	δ_H_ mult. (*J*, Hz)	δ_C_	δ_H_ mult. (*J*, Hz)	δ_C_	δ_H_ mult. (*J*, Hz)	δ_C_
1		132.2		132.1		132.1		131.7
2	7.13 d (2.2)	131.4	7.12 d (2.2)	131.2	7.08 d (2.4)	131.3	7.03 d (2.2)	132.4
3		129.1		128.9		126.6		127.1
4		153.3		153.2		152.1		153.8
5	6.88 d (8.1)	117.0	6.88 d (8.2)	116.9	6.88 d (8.3)	117.5	6.80 d (8.18)	117.1
6	7.00 dd (8.1, 2.2 )	128.9	6.97 dd (8.2, 2.2 )	128.8	7.09 dd (8.3, 2.4 )	129.1	6.96 dd (2.2, 8.18)	129.3
7	3.34 d ( 6.7)	40.1	3.33 d (6.7)	40.0	3.34 brd	39.5	3.23 d (6.7)	40.0
8	5.96 m	139.3	5.96 m	139.2	5.04 m	137.9	6.00 m	139.2
9	5.00 m	115.5	5.02 m	115.4	5.95 m	115.7	4.98 m	115.4
1'		128.6		128.5		132.9		132.5
2'		151.9	7.37 brs	131.4	6.99 brs	130.5	6.95 brs	130.6
3'		127.6	7.37 brs	126.1		126.4		127.8
4'	7.36 d (2.2)	131.3		131.2		149.6		151.5
5'		131.0		131.7		122.3		123.2
6'	7.37 d (2.2)	131.1		151.7	6.96 brs	129.4	6.95brs	129.8
7'	3.51 d (6.7)	35.6		35.4	3.36 brd	39.4		40.9
8'	6.10 m	138.0		137.9	5.11 m	137.6		139.2
9'	5.14 m	115.8		115.8	6.00 m	115.7		115.6
1''		133.2				132.8		134.8
2''	7.09 brs	131.4			7.20 d (8.69)	127.4	7.35 d (2.1)	130.3
3''		131.8			6.70 d (8.69)	115.4		127.1
4''		149.6				155.4		154.4
5''		126.9			6.70 d (8.69)	115.4	6.94 d (8.1)	117.4
6''	7.09 brs	131.4			7.20 d (8.69)	127.4	7.32 dd (8.1, 2.1 )	127.4
7''	3.39 d (7.0)	40.1			5.07 m	78.5	5.12 m	78.7
8''	6.00 m	139.1			a: 2.25 mb: 2.15 m	29.4	a: 2.29 mb: 2.10 m	30.6
9''	5.03 m	115.6			a: 3.04 mb: 2.89 m	25.2	a: 3.08 mb: 2.87 m	26.2
1'''		127.2						132.9
2'''	7.32 d (2.1)	132.0					7.09 d (1.8)	132.7
3'''		130.6						127.1
4'''	7.28 dd (2.1, 8.1)	129.4						153.1
5'''	6.91 d (8.1)	115.8					6.92 d (8.1)	117.4
6'''		155.2					7.05 dd (8.1, 1.8)	129.7
7'''	3.43 d (6.7)	35.1					3.37 d (7.6)	40.1
8'''	6.04 m	138.1					5.90 m	139.1
9'''	5.08 m	115.7					5.03 m	115.5

a: δ (ppm); 400 MHz for ^1^H and 100 MHz for ^13^C; acetone-d6; *J* values (Hz) in parentheses.

b: δ (ppm); 500 MHz for ^1^H and 125 MHz for ^13^C; CDCl_3_; *J* values (Hz) in parentheses.

**Houpulin B (2):** brown syrup; [α]_D_^25^ −7.98 (c 0.7, MeOH); IR (KBr) λ_max_ 3501, 3410, 3074, 1639, 1600, 1504, 1465, 1222, 914, 756 cm^−1^; ^1^H NMR: see Table 1; ^13^C NMR: see Table 1; ESI-MS m/z: 553 ([M+Na]^+^); HR-ESI-MS m/z: 553.2353 ([M+Na]^+^) (Calcd for C_36_H_34_O_4_Na, 553.2355).

**Houpulin C (3):** brown gum; [α]_D_^25^ −65.74 (c 0.11, MeOH); IR (KBr) λ_max_ 3375, 3352, 2924, 1639, 1612, 1496, 1465, 1226, 914, 756 cm^−1^; ^1^H NMR: see Table 1; ^13^C NMR: see Table 1; ESI-MS m/z: 421 ([M+Na]^+^); HR-ESI-MS m/z: 421.1782 ([M+Na]^+^) (Calcd for C_27_H_26_O_3_Na, 421.1780).

**Houpulin D (4):** brown syrup; [α]_D_^25^ −57.64 (c 0.23, MeOH); IR (KBr) λ_max_ 3356, 3340, 2924, 1697, 1608, 1496, 1465, 1226, 914, 756 cm^−1^; ^1^H NMR: see Table 1; ^13^C NMR: see Table 1; ESI-MS m/z: 553 ([M+Na]^+^); HR-ESI-MS m/z: 553.2352 ([M+Na]^+^) (Calcd for C_36_H_34_O_4_Na, 553.2355).

### Preparation of human neutrophils

Blood was taken from healthy human donors (20–30 years old) by venipuncture, using a protocol approved by the Institutional Review Board at Chang Gung Memorial Hospital. All donors gave written consent. The Medical Ethics Committee of Chang Gung Memorial Hospital approved this consent procedure. Neutrophils were isolated with a standard method of dextran sedimentation prior to centrifugation in a Ficoll Hypaque gradient and hypotonic lysis of erythrocytes. Purified neutrophils that contained >98% viable cells, as determined by the trypan blue exclusion method, were re-suspended in calcium (Ca^2+^)-free HBSS buffer at pH 7.4, and were maintained at 4°C before use.

### Measurement of O_2_•^−^ generation

The O_2_•^−^ generation assay was based on the SOD-inhibitable reduction of ferricytochrome *c*. In brief, after supplementation with 0.5 mg/mL ferricytochrome c and 1 mM Ca^2+^, neutrophils (6×10^5^ cells/mL) were equilibrated at 37°C for 2 min and incubated with test compound or an equal volume of vehicle (0.1% DMSO) for 5 min. Cells were activated with 100 nM FMLP during the preincubation of 1 µg/mL cytochalasin B (FMLP/cytochalasin B) for 3 min. Changes in the absorbance with a reduction in ferricytochrome c at 550 nm were continuously monitored in a double-beam, six-cell positioner spectrophotometer with constant stirring (Hitachi U-3010, Tokyo, Japan).

### Measurement of elastase release

Degranulation of azurophilic granules was determined by elastase release as described previously. Experiments were performed using MeO-Suc-Ala-Ala-Pro-Val-*p*-nitroanilide as the elastase substrate. Briefly, after supplementation with MeO-Suc-Ala-Ala-Pro-Val-*p*-nitroanilide (100 µM), neutrophils (6×10^5^/ml) were equilibrated at 37°C for 2 min and incubated with test compound or an equal volume of vehicle (0.1% DMSO) for 5 min. Cells were activated by 100 nM FMLP and 0.5 µg/mL cytochalasin B, and changes in absorbance at 405 nm were continuously monitored to assay elastase release. The results were expressed as the percent of elastase release in the FMLP/cytochalasin B-activated, drug-free control system.

### Statistical analysis

Results were expressed as mean ± S.E.M. Computation of 50% inhibitory concentration (IC_50_) was computer-assisted (PHARM/PCS v.4.2). Statistical comparisons were made between groups using Student's t test. Values of P less than 0.05 were considered to be statistically significant.

## Results

### Characterization of new compounds

**Figure 1:**
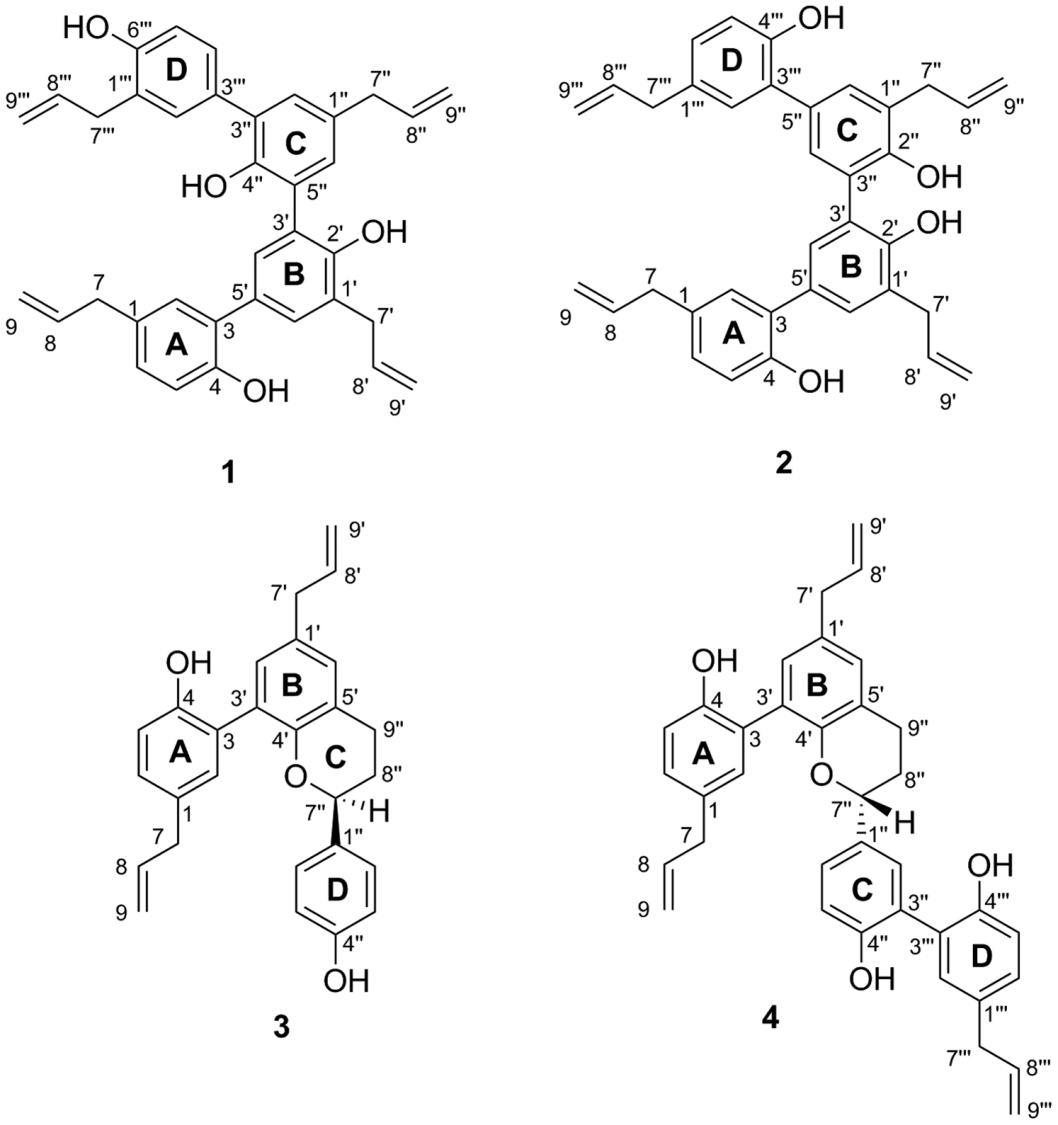
Structures of 1–4.

Repeated silica gel and RP-C_18_ column chromatography of the dichloromethane-soluble fraction from the ethanol extract obtained from the roots of *M. officinalis* afforded four novel oligomeric neolignans (**1–4**) with new carbon skeletons. Compound **1** was obtained as optically active syrup. The positive-mode HR-ESI-MS of **1** showed a sodiated molecular ion peak at *m/z* 553.2357 ([M+Na]^+^), corresponding to a molecular formula of C_36_H_34_O_4_ with 20 indices of hydrogen deficiency (IHD). The absorption bands in the IR spectrum indicated the presence of hydroxy (3502 cm^−1^) and phenyl groups (1639 and 1504 cm^−1^). Analysis of the ^13^C NMR, DEPT135 and HMQC spectral data identified 36 carbon signals consistent with four oxygenated quaternary aromatic carbons at δ 155.2, 153.3, 151.9, and 149.6; eleven tertiary aromatic carbons at δ 132.0, 131.4, 131.4, 131.4, 131.3, 131.1, 131.0, 129.4, 128.9, 117.0 and 115.8; nine quaternary aromatic carbons at δ 133.2, 132.2, 131.8, 130.6, 129.1, 128.6, 127.6, 127.2, and 126.9; eight olefinic carbons at δ 139.3, 139.1, 138.1, 138.0, 115.8, 115.7, 115.6, and 115.5; and four aliphatic methylene carbons at δ 40.1, 40.1, 35.6, and 35.1. The ^1^H NMR spectrum of **1** displayed two sets of ABX-type aromatic signals at δ 6.88 (1H, d, *J* = 8.1 Hz, H-5), 7.00 (1H, dd, *J* = 8.1, 2.2 Hz, H-6), and 7.13 (1H, d, *J* = 2.2 Hz, H-2), as well as 6.91 (1H, d, *J* = 8.1 Hz, H-5′″), 7.28 (1H, dd, *J* = 8.1, 2.1 Hz, H-4′″), and 7.32 (1H, d, *J* = 2.1 Hz, H-2′″). In addition, there were two sets of *meta*-coupled aromatic protons at δ 7.09 (1H, brs, H-2″) and 7.09 (1H, brs, H-6″) and 7.36 (1H, d, *J* = 2.2 Hz, H-4′) and 7.37 (1H, d, *J* = 2.2 Hz, H-6′). Based on the ^1^H-^1^H COSY spectrum, four sets of allyl groups were found at δ 3.34 (2H, d, *J* = 6.7 Hz, H-7), 5.96 (1H, m, H-8), and 5.00 (2H, m, H-9); 3.51 (2H, d, *J* = 6.7 Hz, H-7′), 6.10 (1H, m, H-8′), and 5.14 (2H, m, H-9′); 3.39 (2H, d, *J* = 6.9 Hz, H-7″), 6.00 (1H, m, H-8″), and 5.03 (2H, m, H-9″); and 3.43 (2H, d, *J* = 6.7 Hz, H-7′″), 6.04 (1H, m, H-8′″), and 5.08 (2H, m, H-9′″). From the above spectroscopic data and the proposed biomimetic synthesis in prior studies [17], [18], [19], the chemical structure of **1** should be an *o*,*o*-/*o*,*p*-linked tetramer containing four C_6_-C_3_ subunits (moieties A–D shown in Figure 1). The connections of these moieties were further elucidated via 2D-correlational techniques, including HMBC and NOESY analyses. In the HMBC spectrum, ^2^ *J*, ^3^ *J*-correlations from δ 3.34 (H-7) to δ 128.9 (C-6), 131.4 (C-2), and 132.2 (C-1), from δ 3.51 (H-7′) to δ 128.6 (C-1′), 131.1 (C-6′), and 151.9 (C-2′), and from δ 6.88(H-5), 7.36 (H-4′), and 7.37 (H-6′) to δ 129.1 (C-3) indicated that subunits A and B were linked through C-3/C-5′ similarly to honokiol. In addition, the long range HMBC cross-peaks from δ 3.39 (H-7″) to δ 131.4 (C-2″), 131.4 (C-6″), and 133.2 (C-1″); from δ 3.43 (H-7′″) to δ 127.2 (C-1′″), 132.0 (C-2′″) and 155.2 (C-6′″); from δ 7.28 (H-4′″) to δ 131.8 (C-3″); and from δ 7.09 (H-2″) to δ 130.6(C-3′″) revealed that subunits C and D were also connected similarly to honokiol. The NOE correlations of H-2/H-7, H-7/H-6, OH-4/H-5 in subunit A; H-6′/H-7′ in subunit B; H-2″/H-7″, H-7″/H-6″ in subunit C; H-2′″/H-7′″, OH-6′″/H-5′″ in subunit D; H-2/H-4′ between subunits A and B; and H-4′/H-6″ between subunits B and C established the connectivity of the two honokiol fragments to be C-3′/C-5″, and the structure of **1** was determined conclusively, as shown in Figure 1. Compound **1** was named houpulin A.

The positive-mode HR-ESI-MS of compound **2** displayed a sodiated molecular ion peak at *m/z* 553.2353 ([M+Na]^+^) consistent with a molecular formula of C_36_H_34_O_4_ with 20 degrees of unsaturation, as also found in **1**. The UV absorption maxima and IR absorption bands of **2** were very similar to those of **1**. The ^1^H NMR spectrum of **2** exhibited symmetric proton signals including one set of ABX-type aromatic signals, two overlapped *meta*-coupled signals, and two allyl groups. The HMBC correlations from δ 7.37 (H-6′) to δ 126.1 (C-5′), 128.9 (C-3), 131.2 (C-4′), and 151.7 (C-2′); from δ 3.53 (H-7′) to δ 128.5 (C-1′), 131.4 (C-6′), and 151.7 (C-2′); and from δ 3.33 (H-7) to δ 128.8 (C-6) and 131.2 (C-2) supported the presence of a C-3/C-5′ linkage between subunits A and B, as also found in **1**. However, a comparison of the proton and carbon NMR spectra of **2** with those of **1** suggested that the two honokiol moieties in **2** are connected symmetrically. Thus, the two honokiol fragments are connected between C-3′ and C-3″, rather than C-3′ and C-5″. Therefore, the structure of **2** was elucidated as shown in Figure 1, and the compound has been named houpulin B.

**Figure 2:**
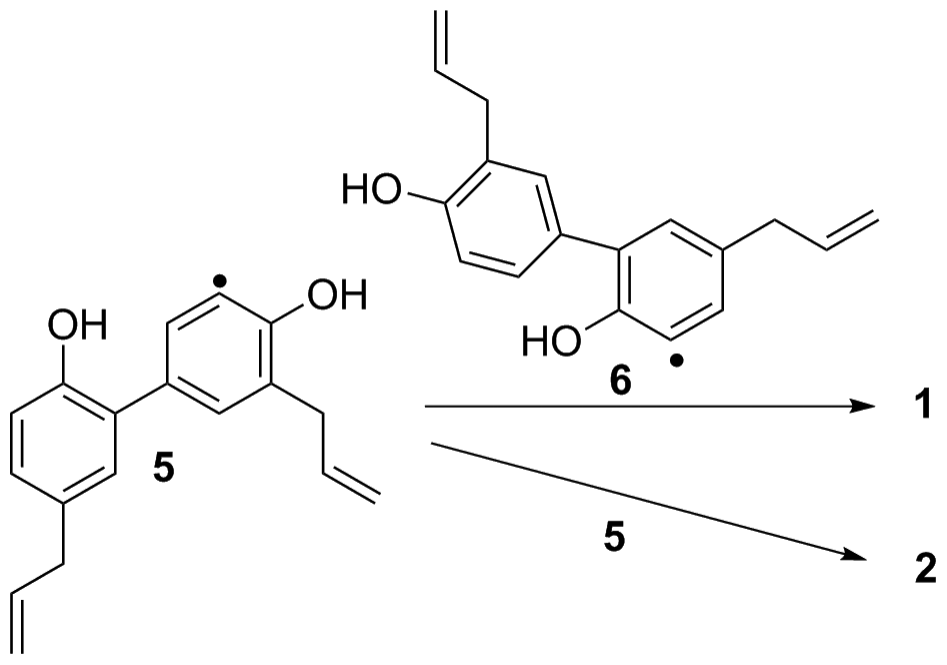
Plausible biosynthetic pathway to 1 and 2.

Figure 2 depicts our proposed biogenetic pathway to compounds **1** and **2**, with the compounds being derived by bimolecular coupling between two honokiol radical derivatives. Because the radical intermediate would be more stable if the radical was located at the *ortho*-position to the hydroxy group, the major honokiol radical intermediates are **5** and **6**, and the resultant coupling products are compounds **1** and **2**, which exhibit the new carbon skeletons characterized in the present work.

Compound **3** was purified as an optically active gum with a molecular formula of C_27_H_26_O_3_, which was deduced by HR-ESI-MS analysis. The IR absorption bands at 3375, 1612, and 1496 cm^-1^ indicated the presence of hydroxy and phenyl groups in **3**. The ^1^H NMR spectrum of **3** exhibited ABX-type aromatic signals at δ 6.88 (1H, d, *J* = 8.3 Hz, H-5), 7.08 (1H, d, *J* = 2.2 Hz, H-2), and 7.09 (1H, dd, *J* = 8.3, 2.2 Hz, H-6); one set of A_2_B_2_ signals at δ 6.70 (2H, d, *J* = 8.7 Hz, H-2″) and 7.20 (2H, d, *J* = 8.7 Hz, H-3″); one set of *meta*-coupled signals at δ 6.96 (1H, br s, H-6′) and 6.99 (1H, br s, H-2′); and two allyl groups at δ 3.34 (2H, brd, H-7), 5.95 (1H, m, H-8), 5.04 (2H, m, H-9), and 3.36 (2H, brd, H-7′), 6.00 (1H, m, H-8′), 5.11 (2H, m, H-9′). In addition, there were five aliphatic proton signals at δ 5.07 (1H, m, H-7″), 2.25 (1H, m, H_a_-8″), 2.15 (1H, m, H_b_-8″), 3.04 (1H, m, H_a_-9″) and 2.89 (1H, m, H_b_-9″). The ^13^C-, DEPT-135, and HSQC NMR spectra revealed 27 carbon signals, three oxygenated quaternary aromatic carbons at δ 149.6, 152.1, and 155.4; nine tertiary aromatic carbons at δ 115.4, 115.4, 117.5, 127.4, 127.4, 129.1, 129.4, 130.5, and 131.1; nine quaternary aromatic carbons at δ 122.3, 126.4, 126.6, 132.2, 132.8, 132.9, 149.6, 152.1, and 155.4; four olefinic carbons at δ 137.9, 137.6, 115.7, and 115.7; one oxygenated aliphatic carbon at δ 78.5; and four methylene aliphatic carbons at δ 25.2, 29.4, 39.4, and 39.5. These spectroscopic data suggested the presence of two neolignan moieties (IHD = 10) and one *p*-disubstituted benzene ring (IHD = 4), which leaves one degree of unsaturation. In the HMBC spectrum, the long-range correlations from δ 6.88 (H-5) to δ 126.6 (C-3), 132.2(C-1), and 152.1 (C-4); from δ 6.99 (1H, br s, H-2′) to δ 39.4 (C-7′), 126.6 (C-3), and 149.6 (C-4′); and from δ 5.07 (1H, m, H-7″) to δ 127.4 (C-2″ and C-6″) indicated that subunits A and B were linked through C-3/C-3′ similarly to magnolol and that subunit C was attached to subunit B between C-4′ and C-5′ to form a dihydrobenzopyran ring. In the NOESY spectrum of **3**, the cross-peaks corresponding to H-7/H-2 and H-6; H-7′/H-2′ and H-6′; H-6′/H-9″; and H-7″/H-2″ and H-6″ allowed for the complete assignment of the proton and carbon signals. Furthermore, compound **3** displayed a negative Cotton effect at 280 nm [20], [21] in the CD spectrum. Therefore, the absolute configuration at C-7″ was assigned as *S*. The structure of **3** was elucidated as shown in Figure 1, and the compound given the name houpulin C.

Compound **4** was obtained as optically active syrup with a molecular formula of C_36_H_34_O_4_, which was determined by a pseudomolecular ion peak at *m/z* 553.2352 in the HR-ESI-MS analysis. The IR spectrum displayed absorption bands at 3356, 1608, and 1496 cm^−1^, which are consistent with the presence of hydroxy and benzyl functionalities, respectively. The ^1^H NMR spectrum of **4** exhibited three sets of ABX-type aromatic signals, three sets of allyl groups, one set of *meta-*coupled protons, and five aliphatic protons, which were very similar to those of compound **3**. From the MS and NMR (^13^C, DEPT135, and HSQC) data, one additional C_6_-C_3_ subunit was present in compound **4**. This postulation was further corroborated by HMBC analysis. In the HMBC spectrum of **4**, ^2^ *J*, ^3^ *J*-correlations from δ 7.03 (H-2) to δ 127.8 (C-3′) and from δ 6.95 (H-2′) to δ 127.1 (C-3) established the C-3/C-3′ connectivity of subunits A and B; correlations from δ 5.12 (H-7″) to δ 130.3 (C-2″) and 134.8 (C-1″) indicated the formation of a dihydrobenzopyran subunit C connected to subunit B at C-4′ and C-5′; and finally, correlations from δ 7.35 (H-2″) to δ 127.1 (C-3′″) and from δ 7.09 (H-2′″) to δ 127.1 (C-3″) showed that the fourth C_6_-C_3_ subunit was attached at the C-3″ position of subunit C. The NOESY cross-peaks corresponding to H-7′/H-6′ and H-6′/H-9″ as well as H-7″/H-2″ and H-6″ confirmed the connectivities of subunits B and C, as well as subunits C and D, respectively. Furthermore, the stereochemical configuration at C-7″ of compound **4** was assigned as *R* based on a positive Cotton effect at 280 nm [20], [21] observed in the CD spectrum. Consequently, the structure of **4** (Figure 1) was established unambiguously, and the compound has been named houpulin D.

### Biological results

Compounds **1**–**4** were evaluated for inhibition of superoxide anion generation and elastase release by human neutrophils in response to FMLP/cytochalasin B [22], and the data are shown in Table 2. Compounds **1**, **2**, and **4** inhibited superoxide anion generation and elastase release in FMLP/cytochalasin B activated human neutrophils in a concentration-dependent manner. Although compound **3** significantly inhibited elastase release with an IC_50_ value of 3.40±0.53 µM, it also induced superoxide generation by human neutrophils. Among the tested compounds, compound **2** demonstrated the most significant inhibition towards superoxide anion generation and elastase release with IC_50_ values of 2.85±0.16 and 2.00±0.50 µM, respectively, compared with the reference compound sorafenib (IC_50_ of 3.23±0.42 and 2.01±0.13 µM for inhibition of superoxide anion generation and elastase release, respectively).

**Table 2:** Inhibitory effects of **1**–**4** on superoxide anion generation and elastase release by human neutrophils in response to FMLP/CB.

compound	IC_50_ (µM)^a^	
	superoxide anion generation	elastase release
**1**	3.2±0.16***	2.3±0.17***
**2**	2.9±0.16***	2.0±0.50***
**3**	-b	3.4±0.53***
**4**	12.7±4.11***	8.7±1.15***
**magnolol**	19.5±1.40***	8.5±2.87***
**honokiol**	-b	5.7±1.30***
sorafenib^c^	3.2±0.42	2.0±0.13

a Concentration necessary for 50% inhibition. Results are presented as the mean ± S.D. (n = 3).

*** P<0.001 compared with the control value.

b Alone induced superoxide generation by human neutrophils.

c Sorafenib, a tyrosine kinase inhibitor, was used as a positive control.

**Figure 3:**
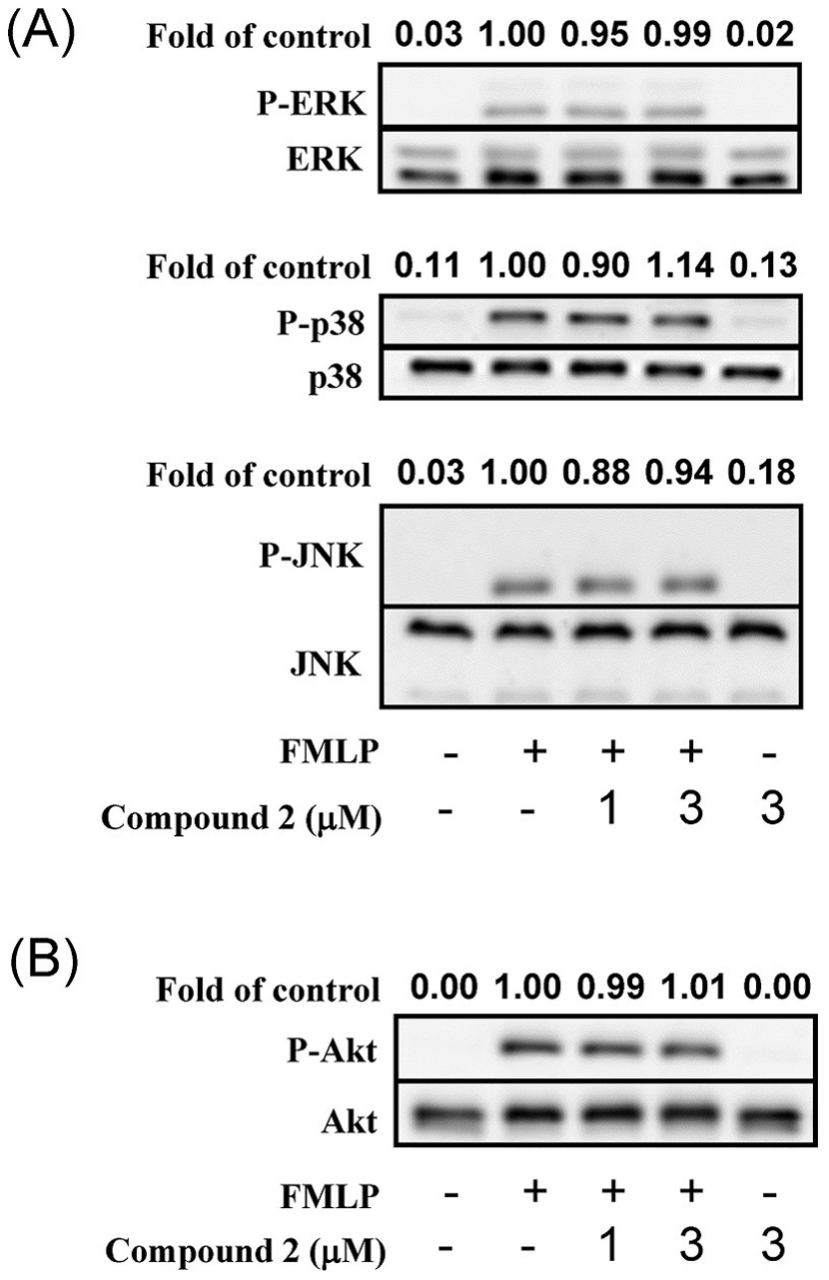
Compound 2 did not alter activation of ERK, p38 MAPK, JNK, and Akt.

**Figure 4:**
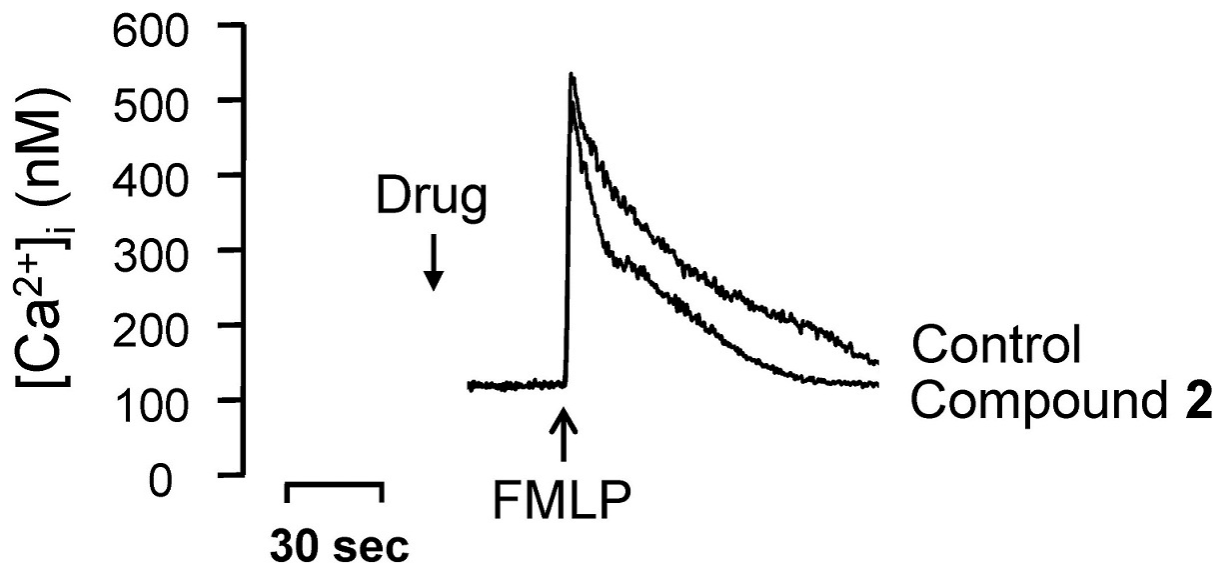
[Ca^2+^]_i_ to return to half of the peak value (*t*_1/2_) by compound 2.

The anti-neutrophilic effect of compound **2** was further evaluated in preliminary mechanistic studies. Compound **2** did not alter activation of ERK, p38 MAPK, JNK, or Akt (Figure 3). Notably, compound **2** failed to alter the peak [Ca^2+^]_i_ values in FMLP-induced cells, but the time it took for [Ca^2+^]_i_ to return to half of the peak value (*t*_1/2_) was significantly shortened by compound **2** (Figure 4). Many neutrophil functions, such as respiratory burst and degranulation, are regulated by calcium signals; thus, calcium clearance mechanisms are increasingly viewed as novel targets for pharmacological control of neutrophilic inflammation [23]. Compound **2** merits further investigation and development as an anti-inflammatory clinical trial candidate.

## Supporting Information

Figure S1 **Significant HMBC and NOE correlations of 1 and 2.**
(TIF)

Figure S2 **Significant HMBC and NOE correlations of 3 and 4.**
(TIF)

Figure S3 **^1^H NMR Spectrum of Houpulin A (1).**
(TIFF)

Figure S4 **^13^C and DEPT135 Spectra of Houpulin A (1).**
(TIFF)

Figure S5 **COSY Spectrum of Houpulin A (1).**
(TIFF)

Figure S6 **NOESY Spectrum of Houpulin A (1).**
(TIFF)

Figure S7 **HSQC Spectrum of Houpulin A (1).**
(TIFF)

Figure S8 **HMBC Spectrum of Houpulin A (1).**
(TIFF)

Figure S9 **IR Spectrum of Houpulin A (1).**
(TIFF)

Figure S10 **Mass Spectrum of Houpulin A (1).**
(TIFF)

Figure S11 **High Resolution Mass Spectrum of Houpulin A (1).**
(TIFF)

Figure S12 **^1^H NMR Spectrum of Houpulin B (2).**
(TIFF)

Figure S13 **^13^C and DEPT135 Spectra of Houpulin B (2).**
(TIFF)

Figure S14 **COSY Spectrum of Houpulin B (2).**
(TIFF)

Figure S15 **NOESY Spectrum of Houpulin B (2).**
(TIFF)

Figure S16 **HMQC Spectrum of Houpulin B (2).**
(TIFF)

Figure S17 **HMBC Spectrum of Houpulin B (2).**
(TIFF)

Figure S18 **IR Spectrum of Houpulin B (2).**
(TIFF)

Figure S19 **Mass Spectrum of Houpulin B (2).**
(TIFF)

Figure S20 **High Resolution Mass Spectrum of Houpulin B (2).**
(TIFF)

Figure S21 **^1^H NMR Spectrum of Houpulin C (3).**
(TIFF)

Figure S22 **^13^C and DEPT135 Spectra of Houpulin C (3).**
(TIFF)

Figure S23 **COSY Spectrum of Houpulin C (3).**
(TIFF)

Figure S24 **NOESY Spectrum of Houpulin C (3).**
(TIFF)

Figure S25 **HMQC Spectrum of Houpulin C (3).**
(TIFF)

Figure S26 **HMBC Spectrum of Houpulin C (3).**
(TIFF)

Figure S27 **IR Spectrum of Houpulin C (3).**
(TIFF)

Figure S28 **CD Spectrum of Houpulin C (3).**
(TIFF)

Figure S29 **Mass Spectrum of Houpulin C (3).**
(TIFF)

Figure S30 **High Resolution Mass Spectrum of Houpulin C (3).**
(TIFF)

Figure S31 **^1^H NMR Spectrum of Houpulin D (4).**
(TIFF)

Figure S32 **^13^C and DEPT135 Spectra of Houpulin D (4).**
(TIFF)

Figure S33 **COSY Spectrum of Houpulin D (4).**
(TIFF)

Figure S34 **NOESY Spectrum of Houpulin D (4).**
(TIFF)

Figure S35 **HSQC Spectrum of Houpulin D (4).**
(TIFF)

Figure S36 **IR Spectrum of Houpulin D (4).**
(TIFF)

Figure S37 **CD Spectrum of Houpulin D (4).**
(TIFF)

Figure S38 **Mass Spectrum of Houpulin D (4).**
(TIFF)

Figure S39 **High Resolution Mass Spectrum of Houpulin D (4).**
(TIFF)

## References

[1] Nakazawa T, Yasuda T, Ohsawa K (2003) Metabolites of orally administered Magnolia officinalis extract in rats and man and its antidepressant-like effects in mice. J Pharm Pharmacol 55: 1583–1591. 14713371 10.1211/0022357022188

[2] Wang Y, Kong L, Chen Y (2005) Behavioural and biochemical effects of fractions prepared from Banxia Houpu decoction in depression models in mice. Phytother Res 19: 526–529. 16114088 10.1002/ptr.1697

[3] Konoshima T, Kozuka M, Tokuda H, Nishino H, Iwashima A, et al (1991) Studies on inhibitors of skin tumor promotion, IX. Neolignans from Magnolia officinalis. J Nat Prod 54: 816–822. 1659613 10.1021/np50075a010

[4] Yahara S, Nishiyori T, Kohda A, Nohara T, Nishioka I (1991) Isolation and characterization of phenolic compounds from Magnoliae cortex produced in China. Chem Pharm Bull 39: 2024–2036.

[5] Syu WJ, Shen CC, Lu JJ, Lee GH, Sun CM (2004) Antimicrobial and cytotoxic activities of neolignans from Magnolia officinalis. Chem. Biodiversity 1: 530–537. 10.1002/cbdv.200490046 17191867

[6] Youn UJ, Chen QC, Jin WY, Lee IS, Kim HJ, et al (2007) Cytotoxic lignans from the stem bark of Magnolia officinalis. J Nat Prod 70: 1687–1689. 17918910 10.1021/np070388c

[7] Shen CC, Ni CL, Shen YC, Huang YL, Kuo CH, et al (2009) Phenolic constituents from the stem bark of Magnolia officinalis. J Nat Prod 72: 168–171. 19086868 10.1021/np800494e

[8] Bae EA, Han MJ, Kim NJ, Kim DH (1998) Anti-Helicobacter pylori activity of herbal medicines. Biol Pharm Bull 21: 990–992. 9781854 10.1248/bpb.21.990

[9] Hu Y, Qiao J, Zhang X, Ge C (2011) Antimicrobial effect of Magnolia officinalis extract against Staphylococcus aureus. J Sci Food Agric 91: 1050–1056. 21384366 10.1002/jsfa.4280

[10] Yang SE, Hsieh MT, Tsai TH, Hsu SL (2003) Effector mechanism of magnolol-induced apoptosis in human lung squamous carcinoma CH27 cells. Br J Pharmacol 138: 193–201. 12522090 10.1038/sj.bjp.0705024 PMC1573650

[11] Vaid M, D.Sharma S, K.Katiyar S (2010) Honokiol, a phytochemical from the Magnolia plant, inhibits photocarcinogenesis by targeting UVB-induced inflammatory mediators and cell cycle regulators: development of topical formulation. Carcinogenesis 31: 2004–2011. 20823108 10.1093/carcin/bgq186

[12] Shigemura K, Arbiser JL, Sun SY, Zayzafoon M, Johnstone PA, et al (2007) Honokiol, a natural plant product, inhibits the bone metastatic growth of human prostate cancer cells. Cancer 109: 1279–1289. 17326044 10.1002/cncr.22551

[13] Chen CR, Tan R, Qu WM, Wu Z, Wang Y, et al (2011) Magnolol, a major bioactive constituent of the bark of Magnolia officinalis, exerts anti-epileptic effects via GABA/benzodiazepine receptor complex in mice. Br J Pharmacol 164: 1534–1546. 21518336 10.1111/j.1476-5381.2011.01456.x PMC3221106

[14] Ho KY, Tsai CC, Chen CP, Huang JS, Lin CC (2001) Antimicrobial activity of honokiol and magnolol isolated from Magnolia officinalis. Phytother. Res. 15: 139–141. 10.1002/ptr.736 11268114

[15] Chao LK, Liao PC, Ho CL, Wang EI, Chuang CC, et al (2010) Anti-inflammatory bioactivities of honokiol through inhibition of protein kinase C, mitogen-activated protein kinase, and the NF-κB pathway to reduce LPS-induced TNFα and NO expression. J Agric Food Chem 58: 3472–3478. 20192217 10.1021/jf904207m

[16] Munroe ME, Businga TR, Kline JN, Bishop GA (2010) Anti-inflammatory effects of the neurotransmitter agonist honokiol in a mouse model of allergic asthma. J Immunol 185: 5586–5597. 20889543 10.4049/jimmunol.1000630 PMC3197781

[17] Kouno I, Hashimoto A, Kawano N, Yang CS (1989) New sesqui-neolignan from the pericarps of Illicium macranthum. Chem Pharm Bull 37: 1291–1292.

[18] Kouno I, Iwamoto C, Kameda Y, Tanaka T, Yang CS (1994) A new triphenyl-type neolignan and a biphenylneolignan from the bark of Illicium simonsii. Chem Pharm Bull 42: 112–114.

[19] Sy LK, Brown GD (1998) Biomimetic synthesis of Illicium oligomeric neolignans. J Chem Res. Synopses 8: 476–477.

[20] Yang Y, Zhang T, Xiao L, Chen RY (2010) Two novel flavanes from the leaves of Morus alba L. J Asian Nat Prod Res. 12: 194–198. 10.1080/10286020903501577 20390764

[21] Antus S, Kurtan T, Juhasz L, Kiss L, Hollosi M, et al (2001) Chiroptical properties of 2,3-dihydrobenzo[b]furan and chromane chromophores in naturally occurring O-heterocycles. Chirality 13: 493–506. 11466774 10.1002/chir.1067

[22] Yang ML, Kuo PC, Hwang TL, Chiou WF, Qian K, et al (2011) Synthesis, in vitro anti-inflammatory and cytotoxic evaluation, and mechanism of action studies of 1-benzoyl-β-carboline and 1-benzoyl-3-carboxy-β-carboline derivatives. Bioorg Med Chem 19: 1674–1682. 21316977 10.1016/j.bmc.2011.01.034

[23] Tintinger GR, Steel HC, Theron AJ, Anderson R (2009) Pharmacological control of neutrophil-mediated inflammation: strategies targeting calcium handling by activated polymorphonuclear leukocytes. Drug Des Devel Ther 2: 95–104. PMC2761182 19920897

