# Supplementary figures and images for: Honokiol Dimers and Magnolol Derivatives with New Carbon Skeletons from the Roots of Magnolia officinalis and Their Inhibitory Effects on Superoxide Anion Generation and Elastase Release
Source: PLoS One. 2013 May 7;8(5):e59502. doi: 10.1371/journal.pone.0059502 (PMC3646836; doi:10.1371/journal.pone.0059502)

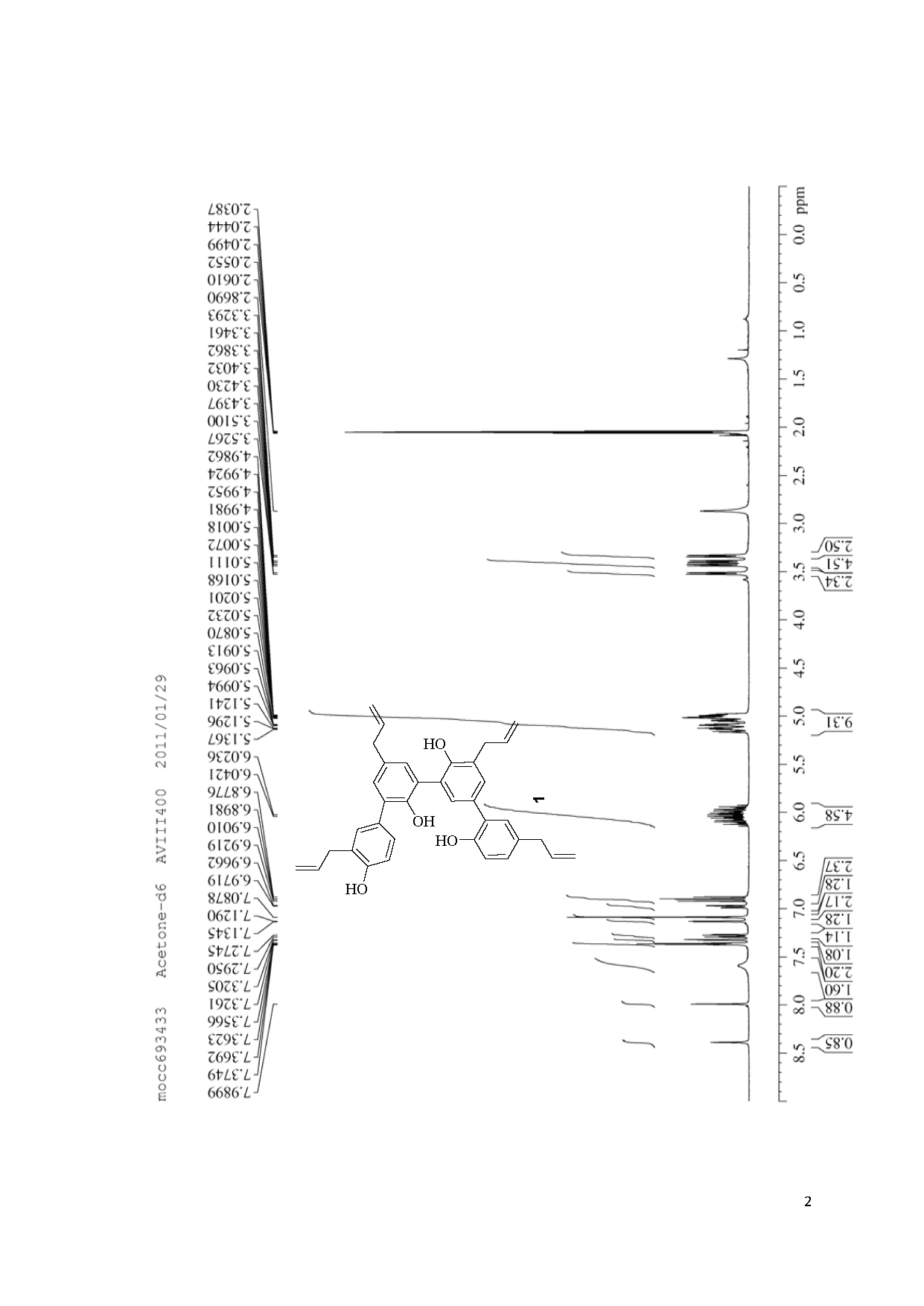

Supplement: Figure S3 — 1H NMR Spectrum of Houpulin A (1). (TIFF) [file pone.0059502.s003.tiff]

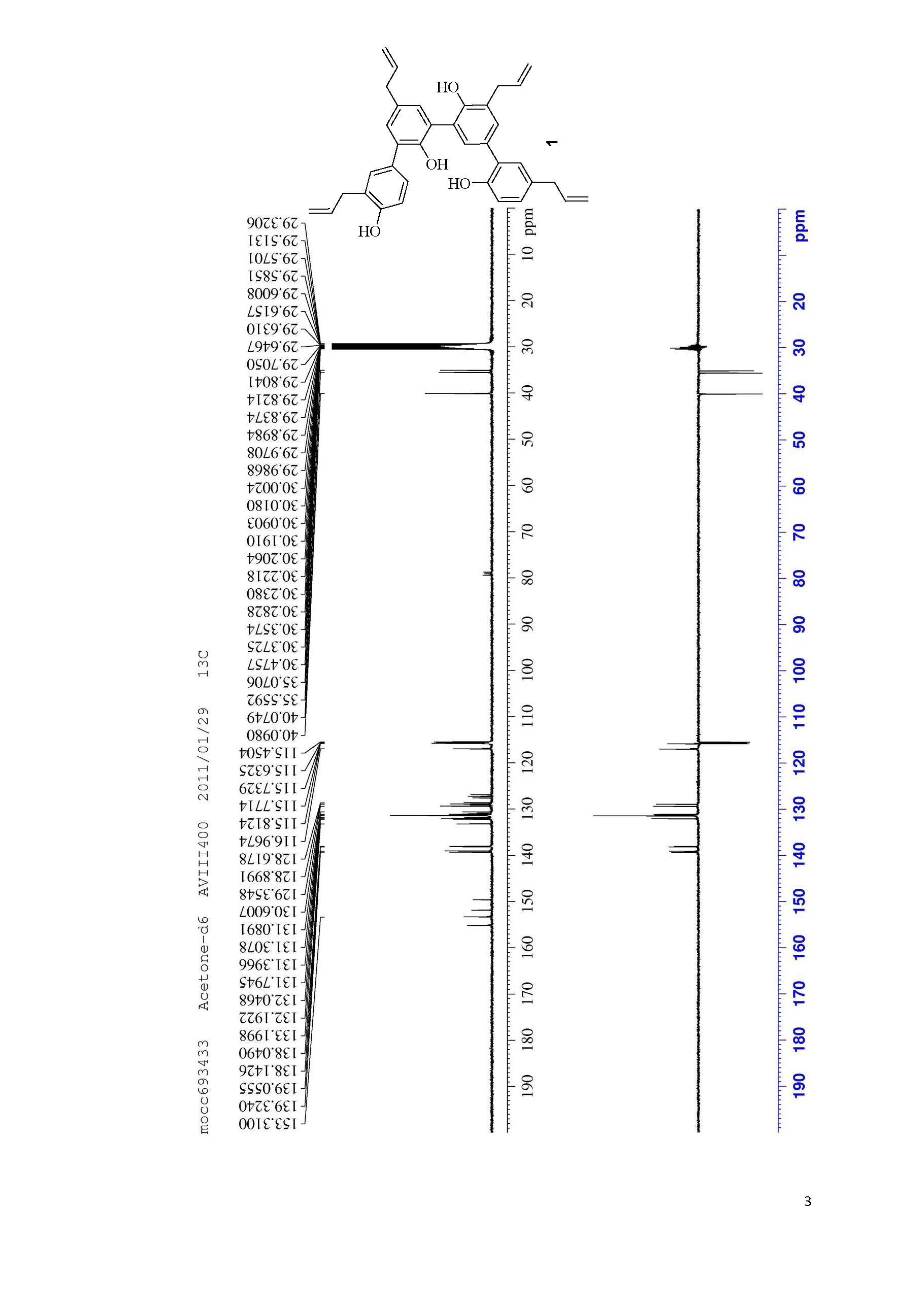

Supplement: Figure S4 — 13C and DEPT135 Spectra of Houpulin A (1). (TIFF) [file pone.0059502.s004.tiff]

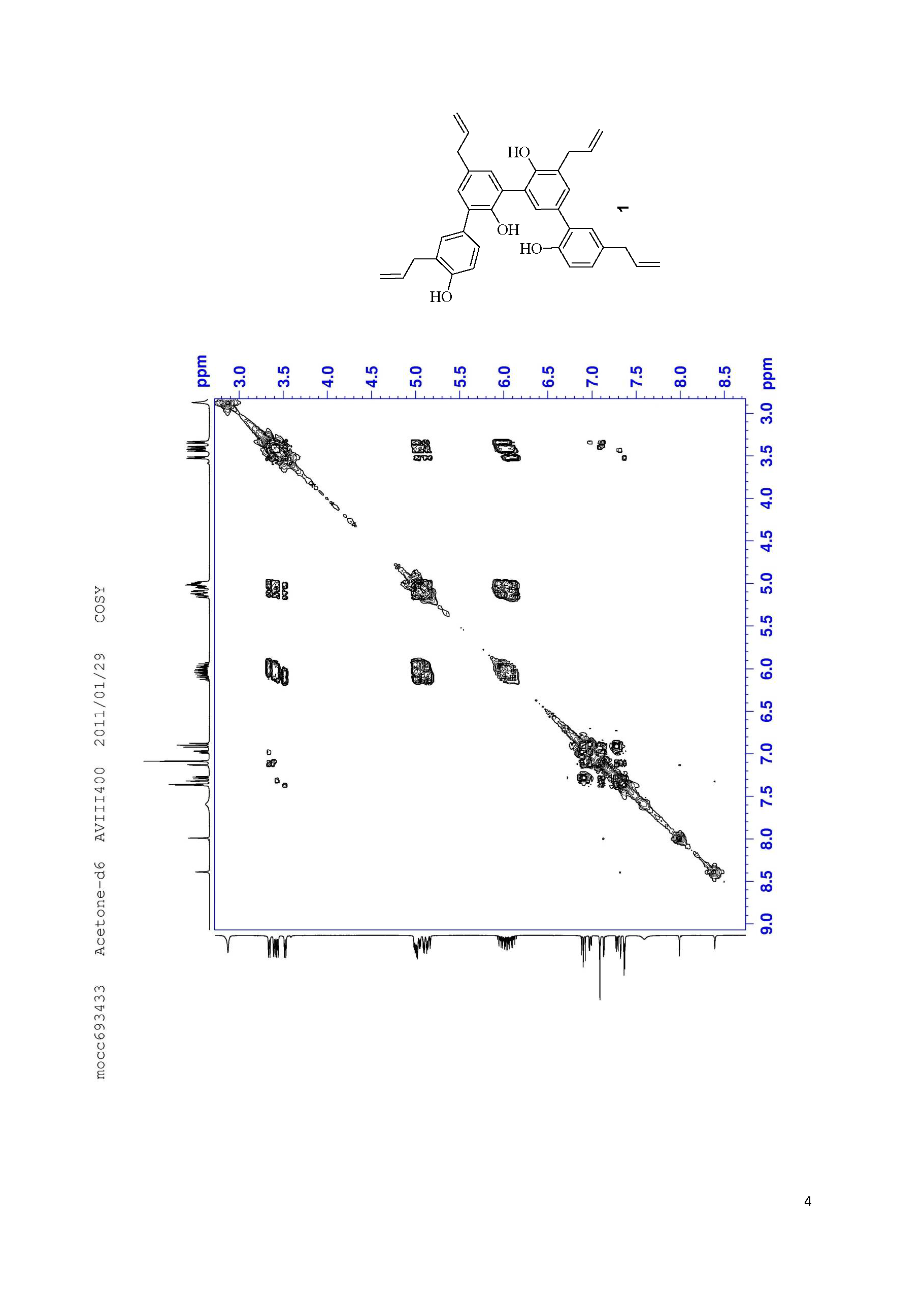

Supplement: Figure S5 — COSY Spectrum of Houpulin A (1). (TIFF) [file pone.0059502.s005.tiff]

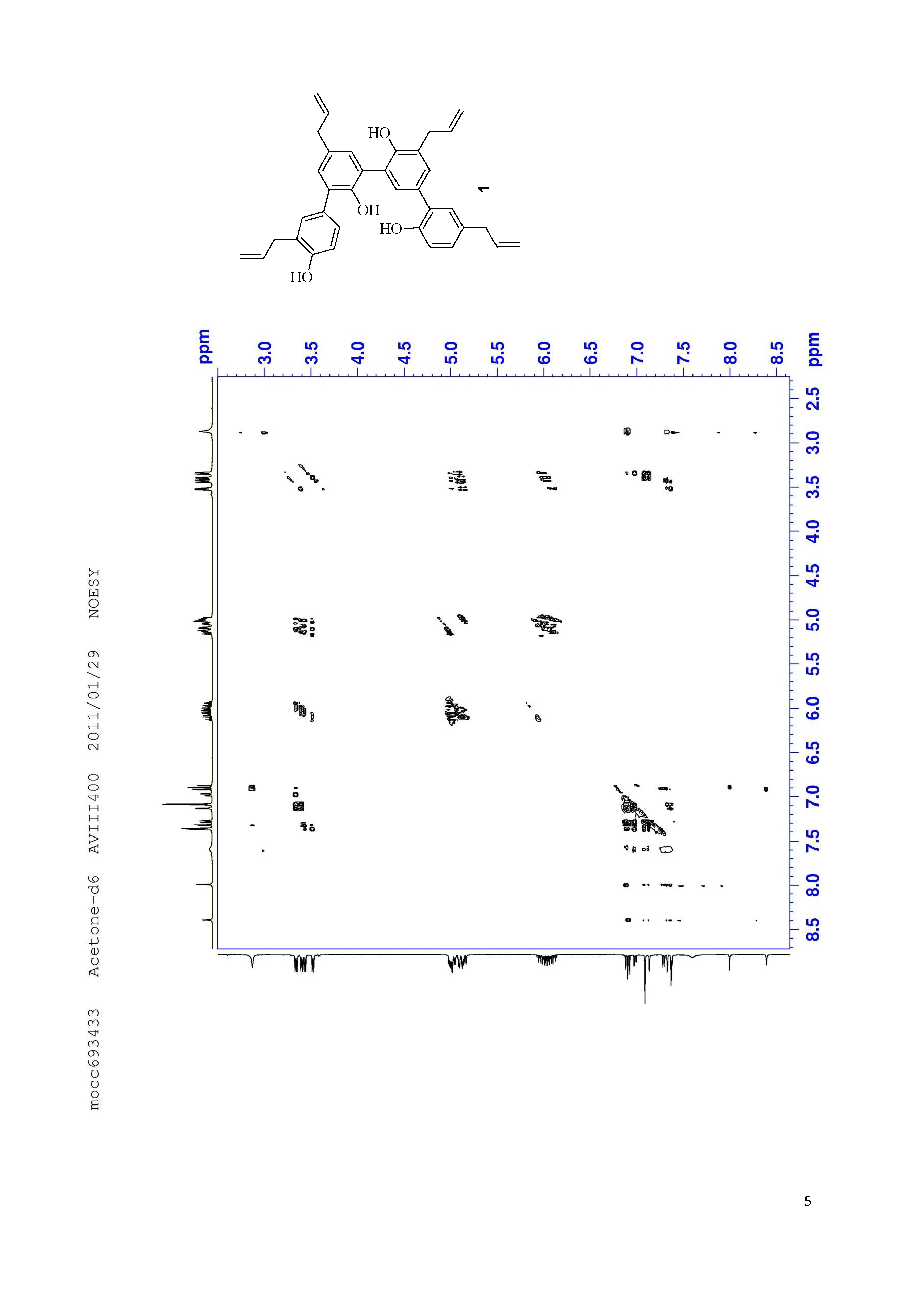

Supplement: Figure S6 — NOESY Spectrum of Houpulin A (1). (TIFF) [file pone.0059502.s006.tiff]

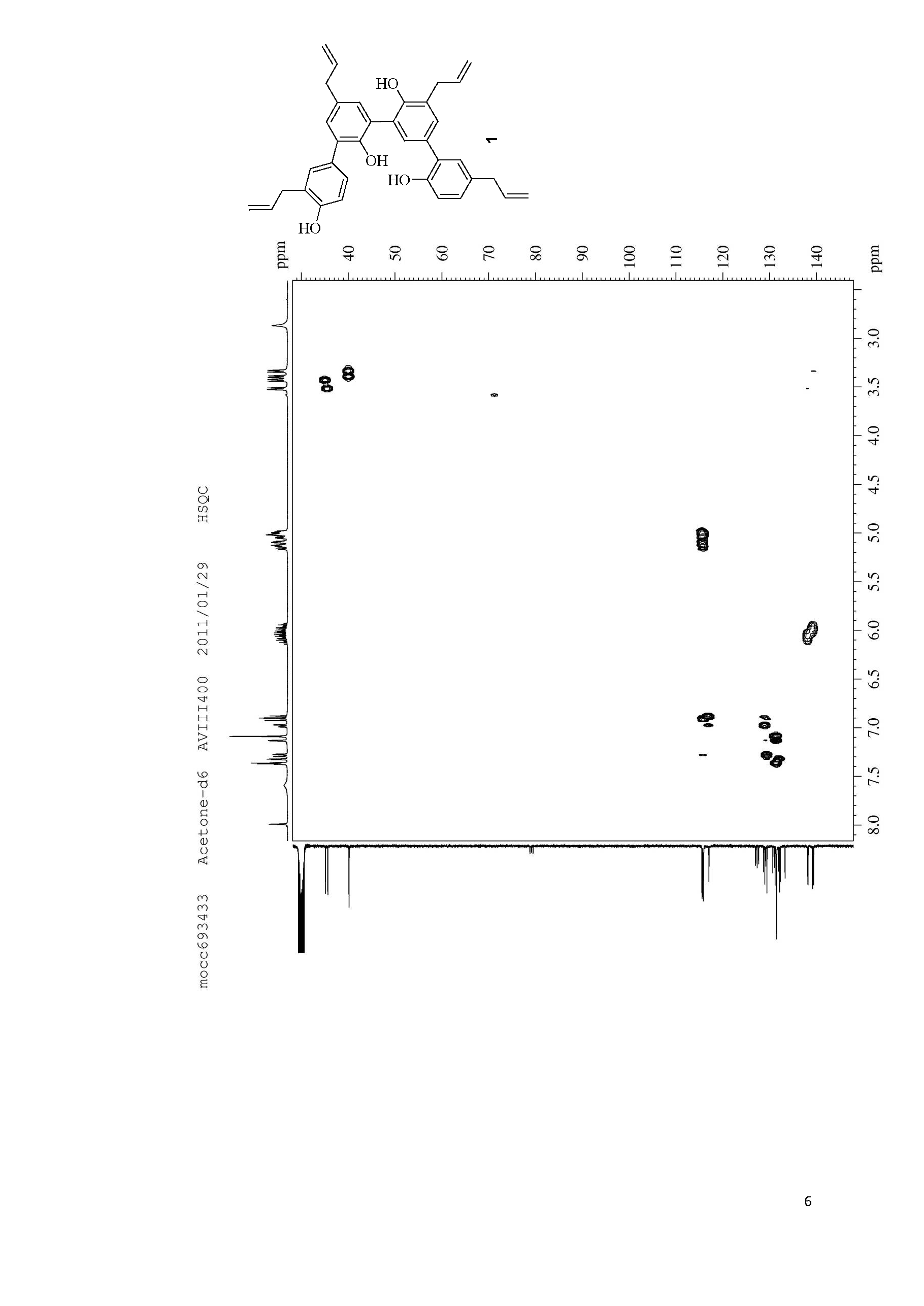

Supplement: Figure S7 — HSQC Spectrum of Houpulin A (1). (TIFF) [file pone.0059502.s007.tiff]

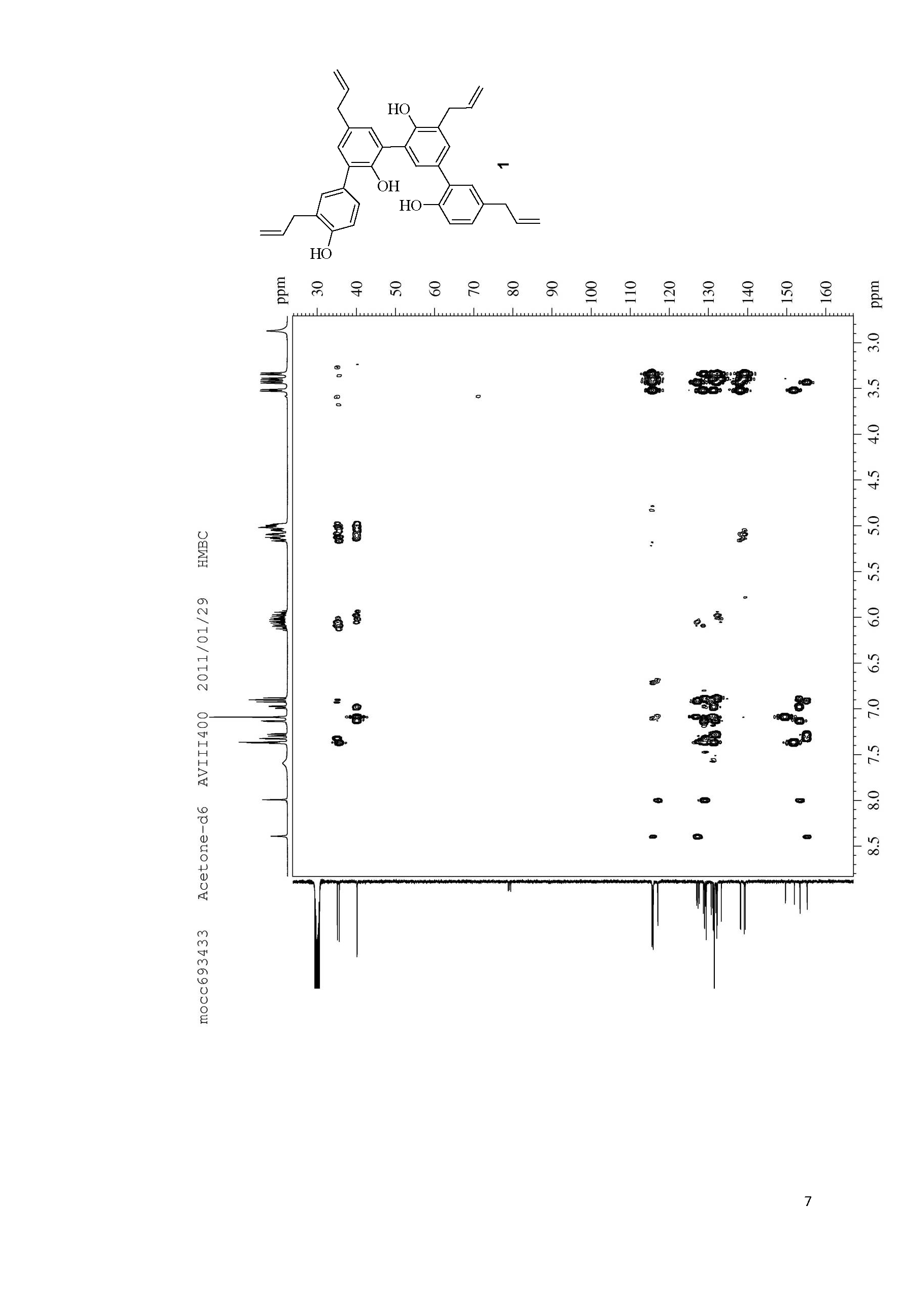

Supplement: Figure S8 — HMBC Spectrum of Houpulin A (1). (TIFF) [file pone.0059502.s008.tiff]

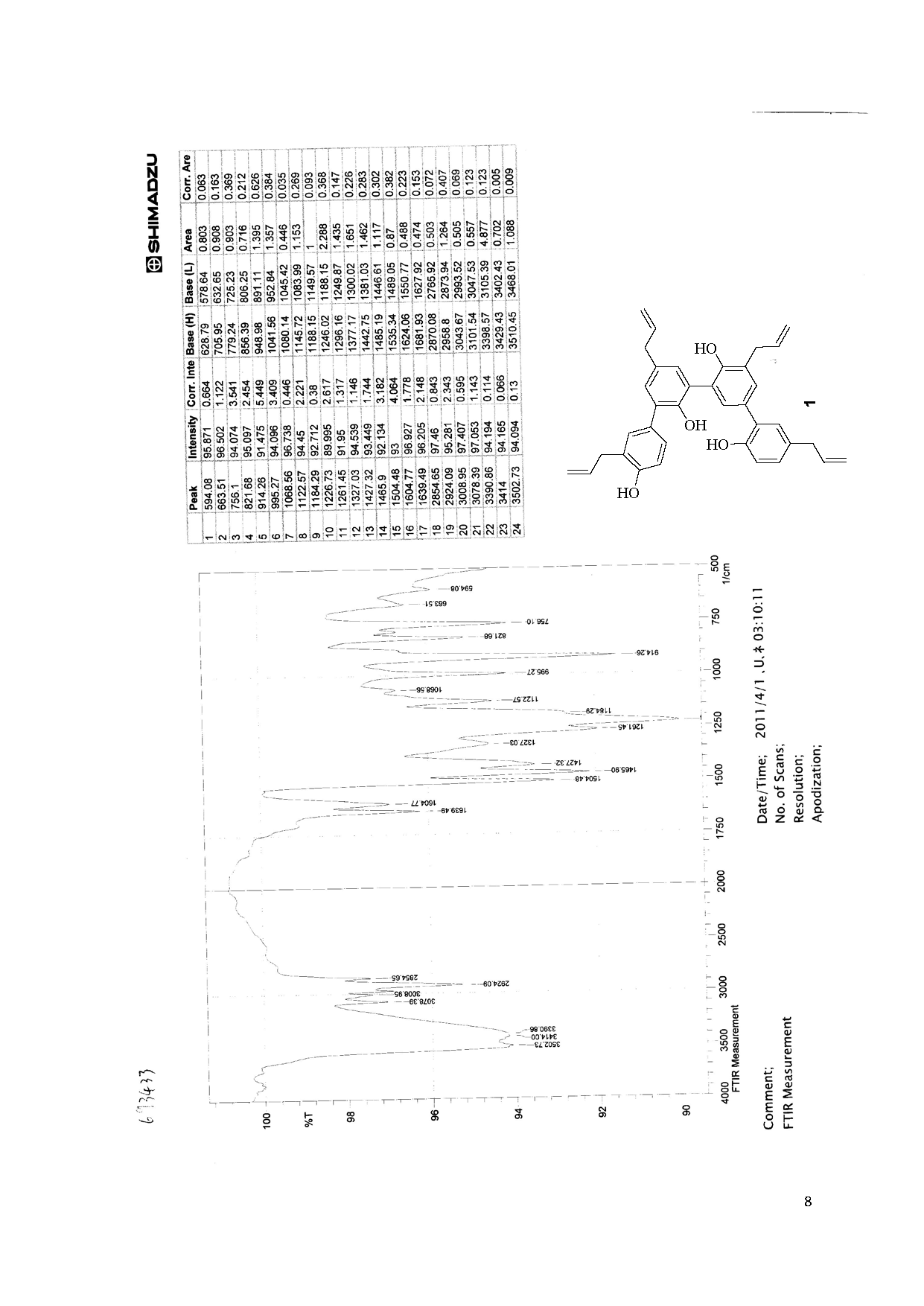

Supplement: Figure S9 — IR Spectrum of Houpulin A (1). (TIFF) [file pone.0059502.s009.tiff]

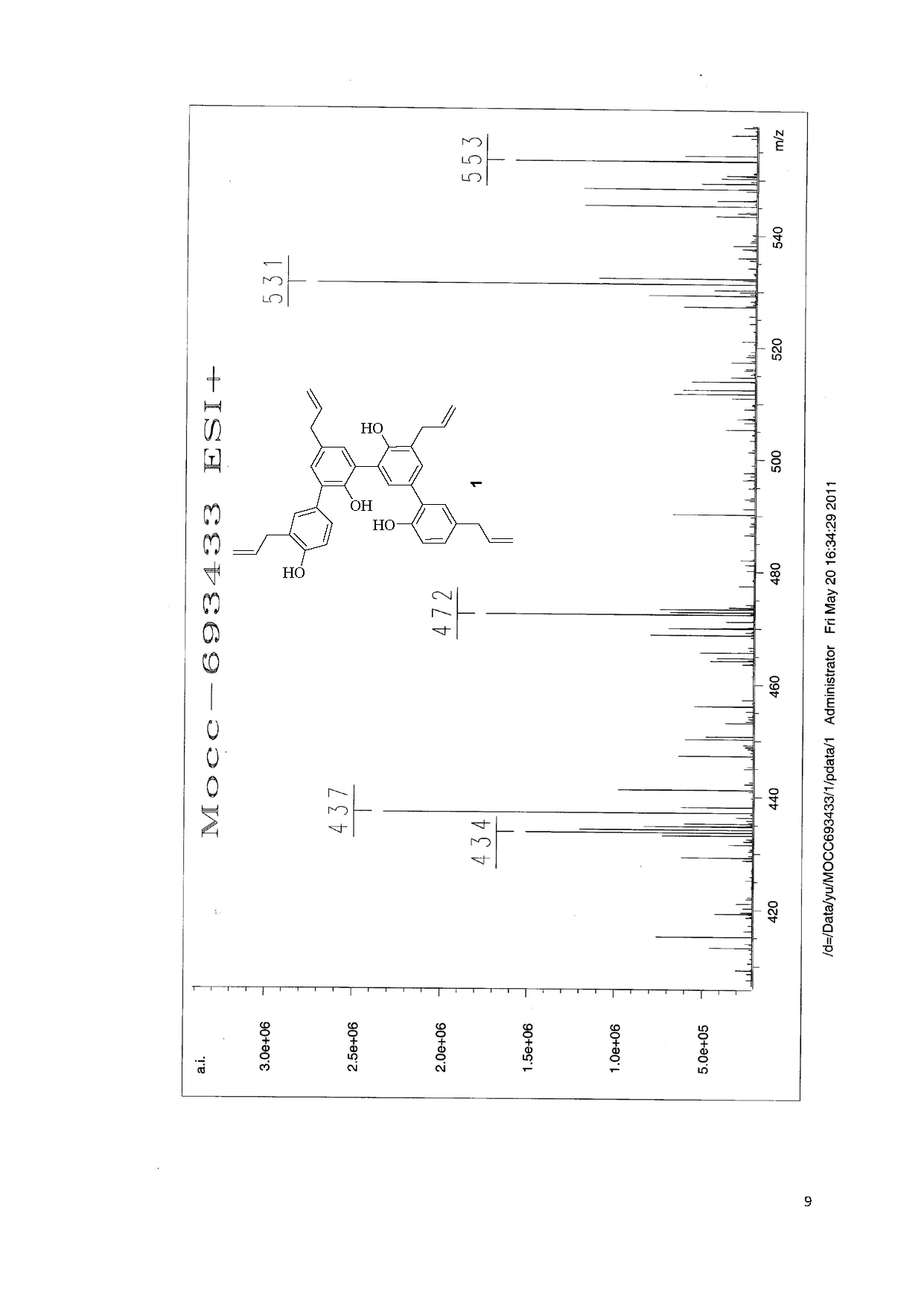

Supplement: Figure S10 — Mass Spectrum of Houpulin A (1). (TIFF) [file pone.0059502.s010.tiff]

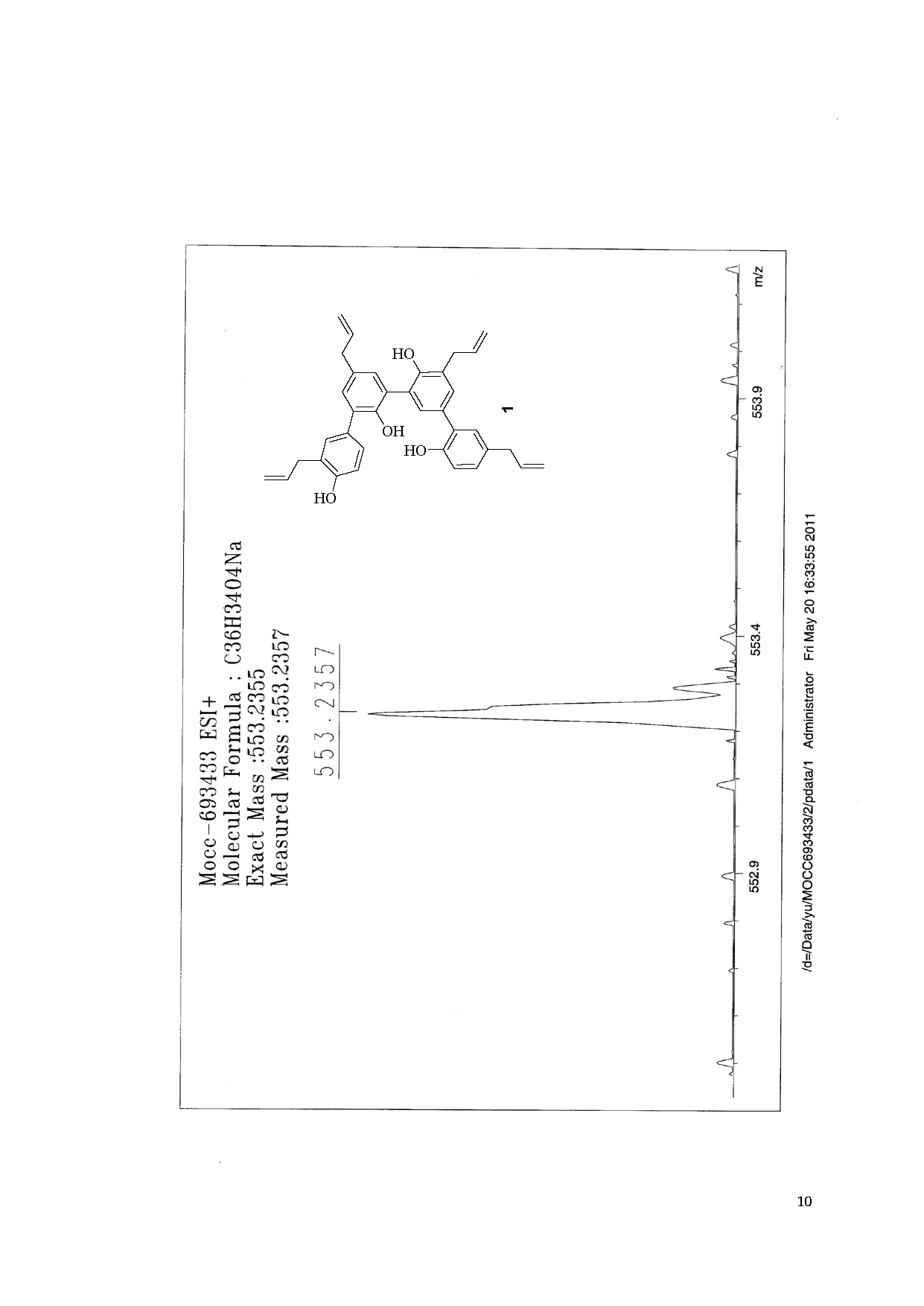

Supplement: Figure S11 — High Resolution Mass Spectrum of Houpulin A (1). (TIFF) [file pone.0059502.s011.tiff]

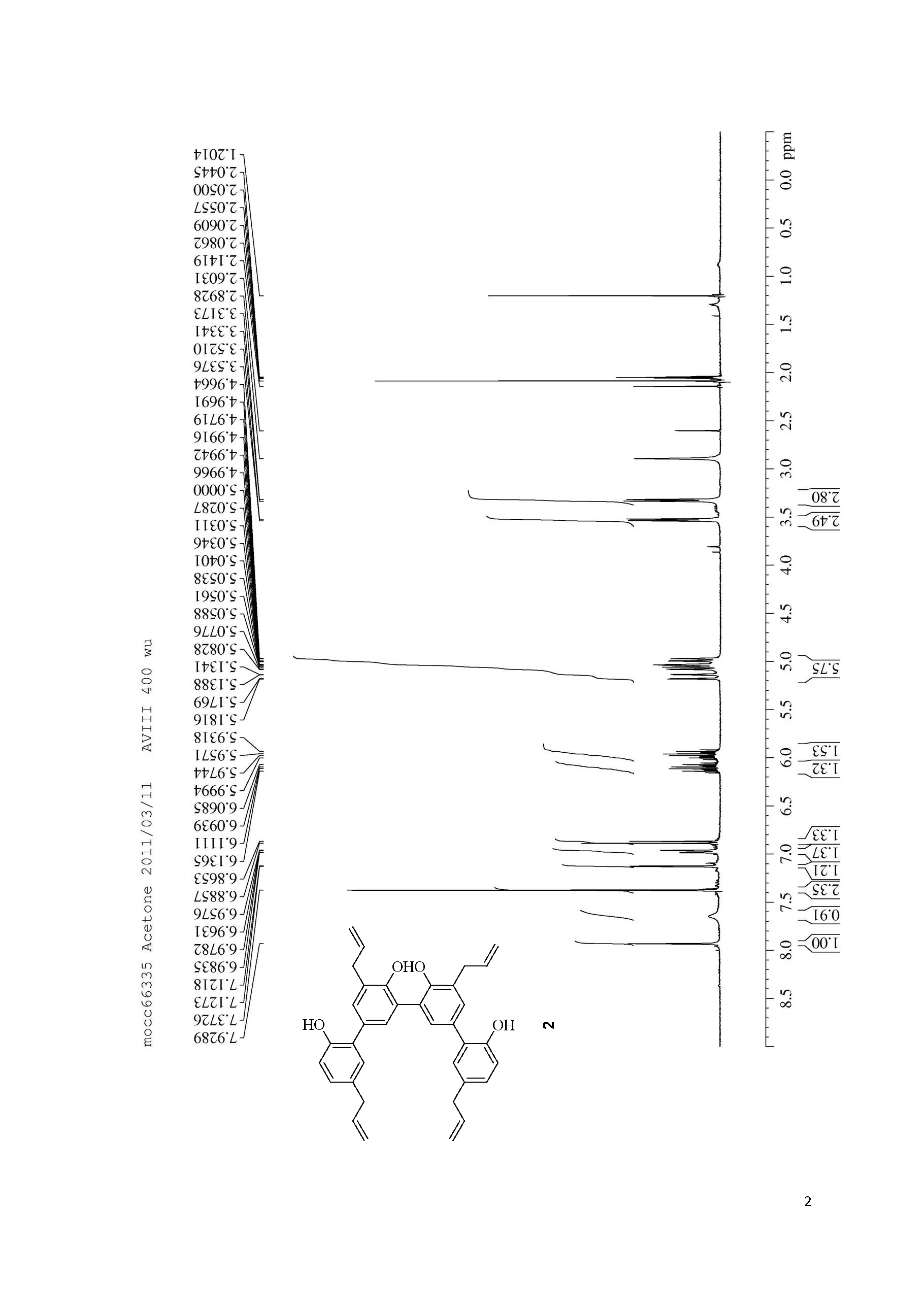

Supplement: Figure S12 — 1H NMR Spectrum of Houpulin B (2). (TIFF) [file pone.0059502.s012.tiff]

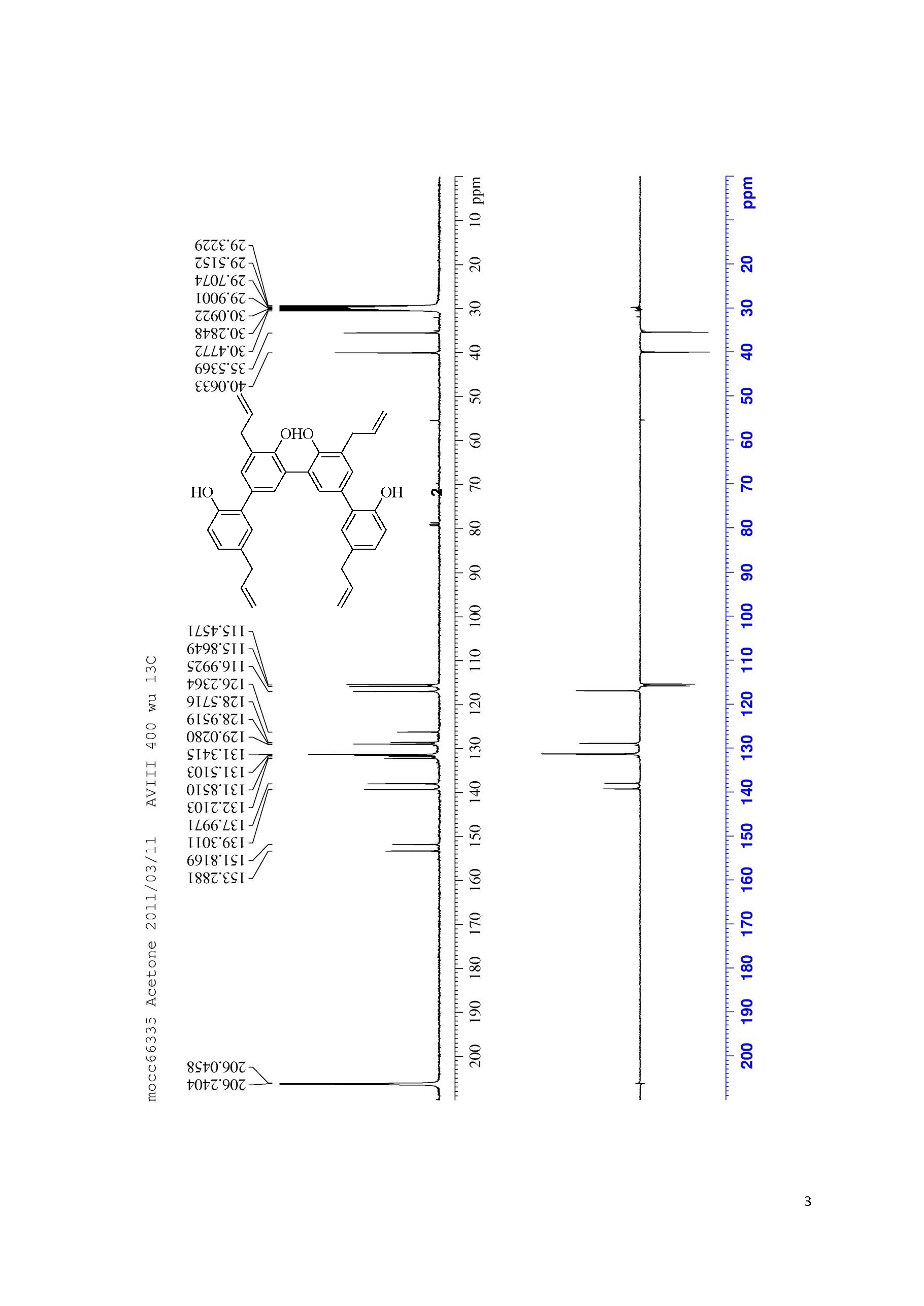

Supplement: Figure S13 — 13C and DEPT135 Spectra of Houpulin B (2). (TIFF) [file pone.0059502.s013.tiff]

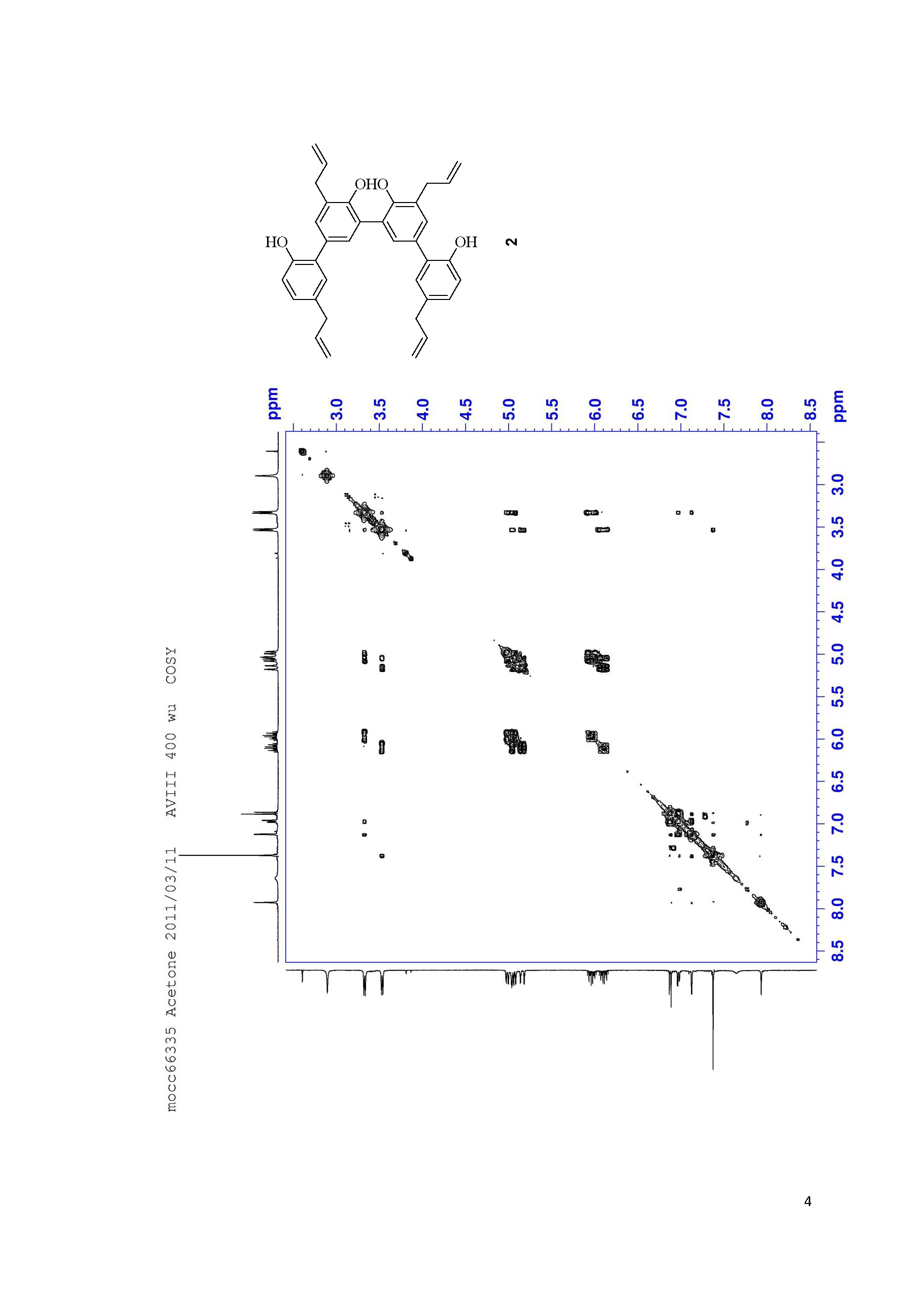

Supplement: Figure S14 — COSY Spectrum of Houpulin B (2). (TIFF) [file pone.0059502.s014.tiff]

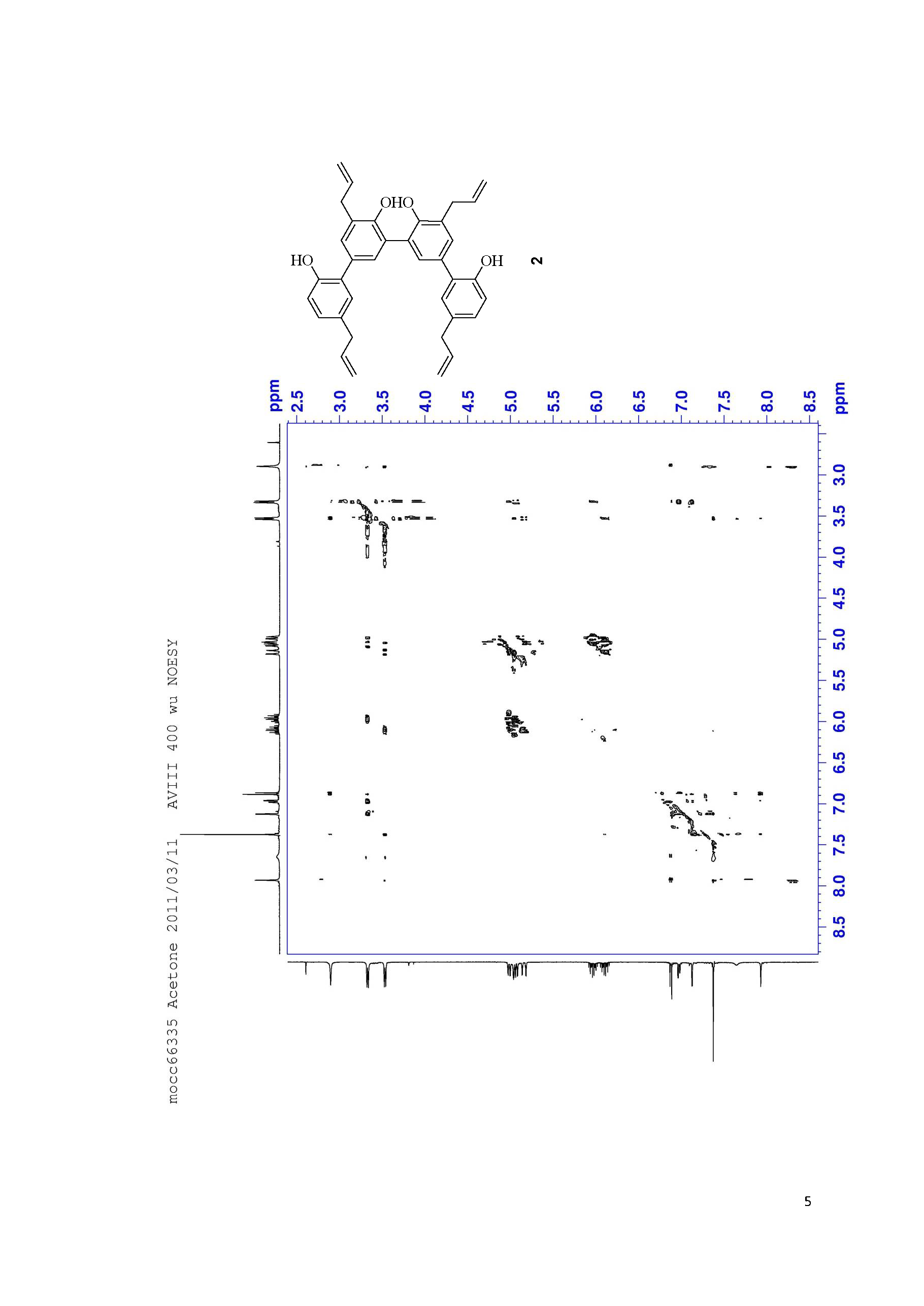

Supplement: Figure S15 — NOESY Spectrum of Houpulin B (2). (TIFF) [file pone.0059502.s015.tiff]

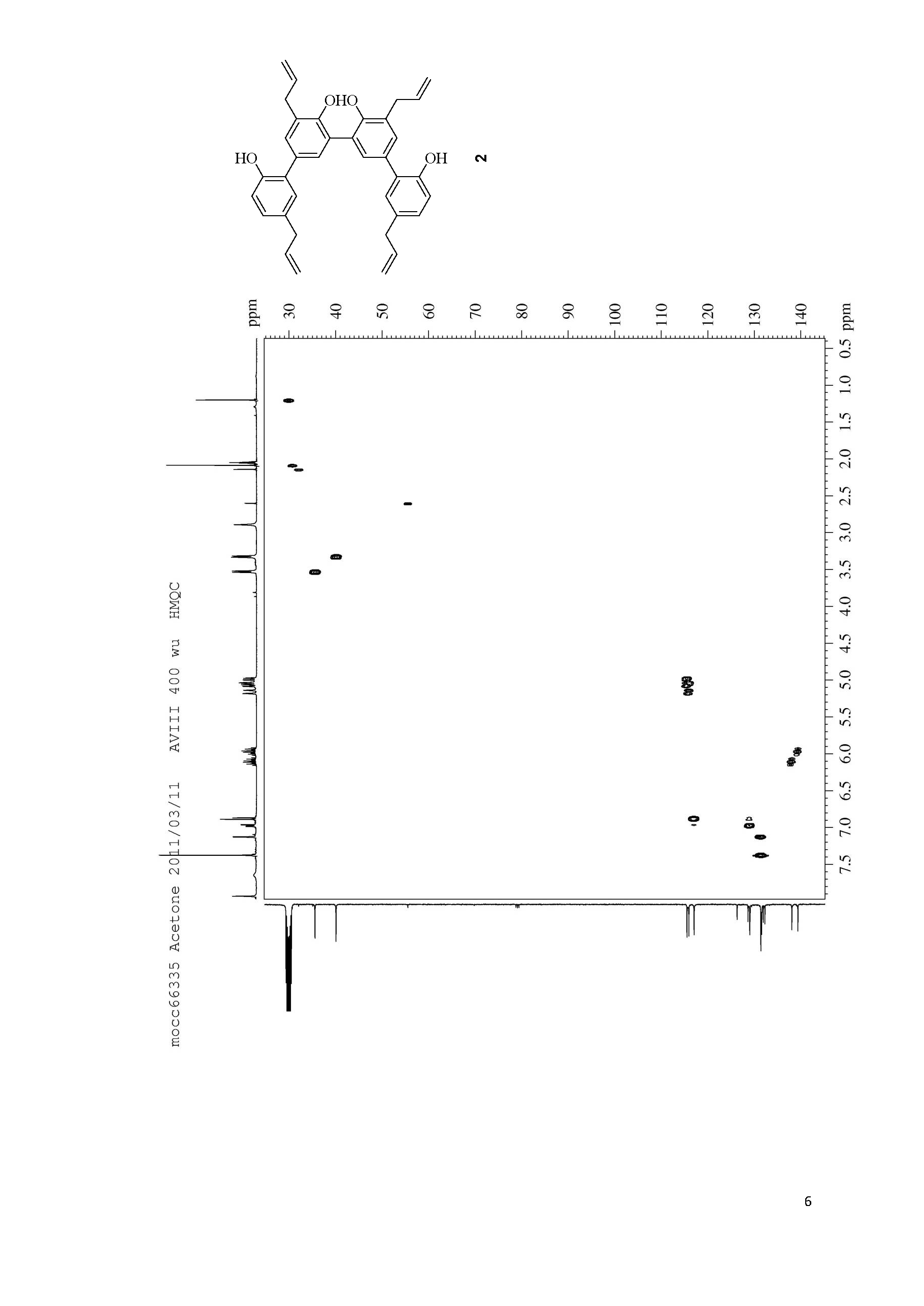

Supplement: Figure S16 — HMQC Spectrum of Houpulin B (2). (TIFF) [file pone.0059502.s016.tiff]

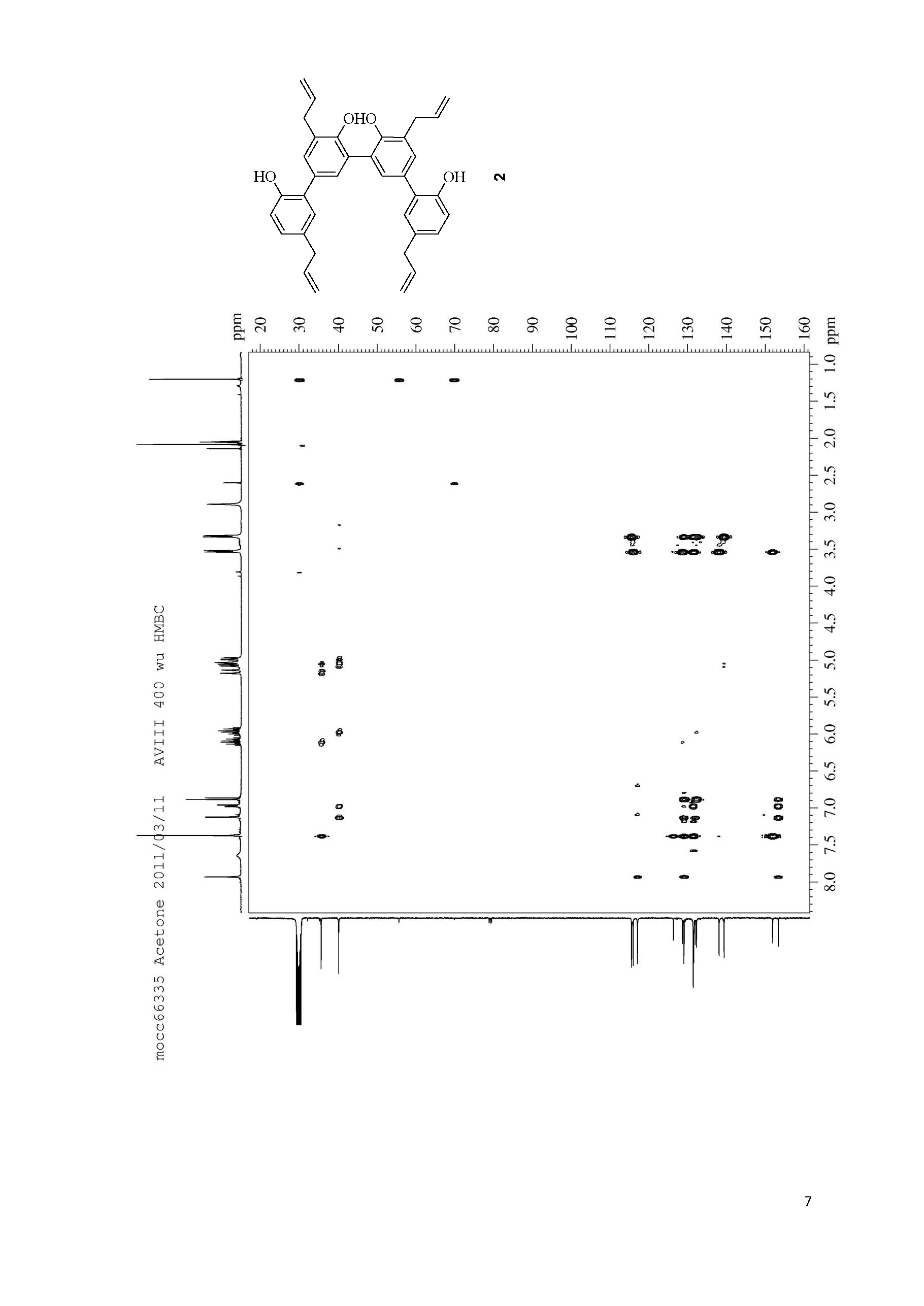

Supplement: Figure S17 — HMBC Spectrum of Houpulin B (2). (TIFF) [file pone.0059502.s017.tiff]

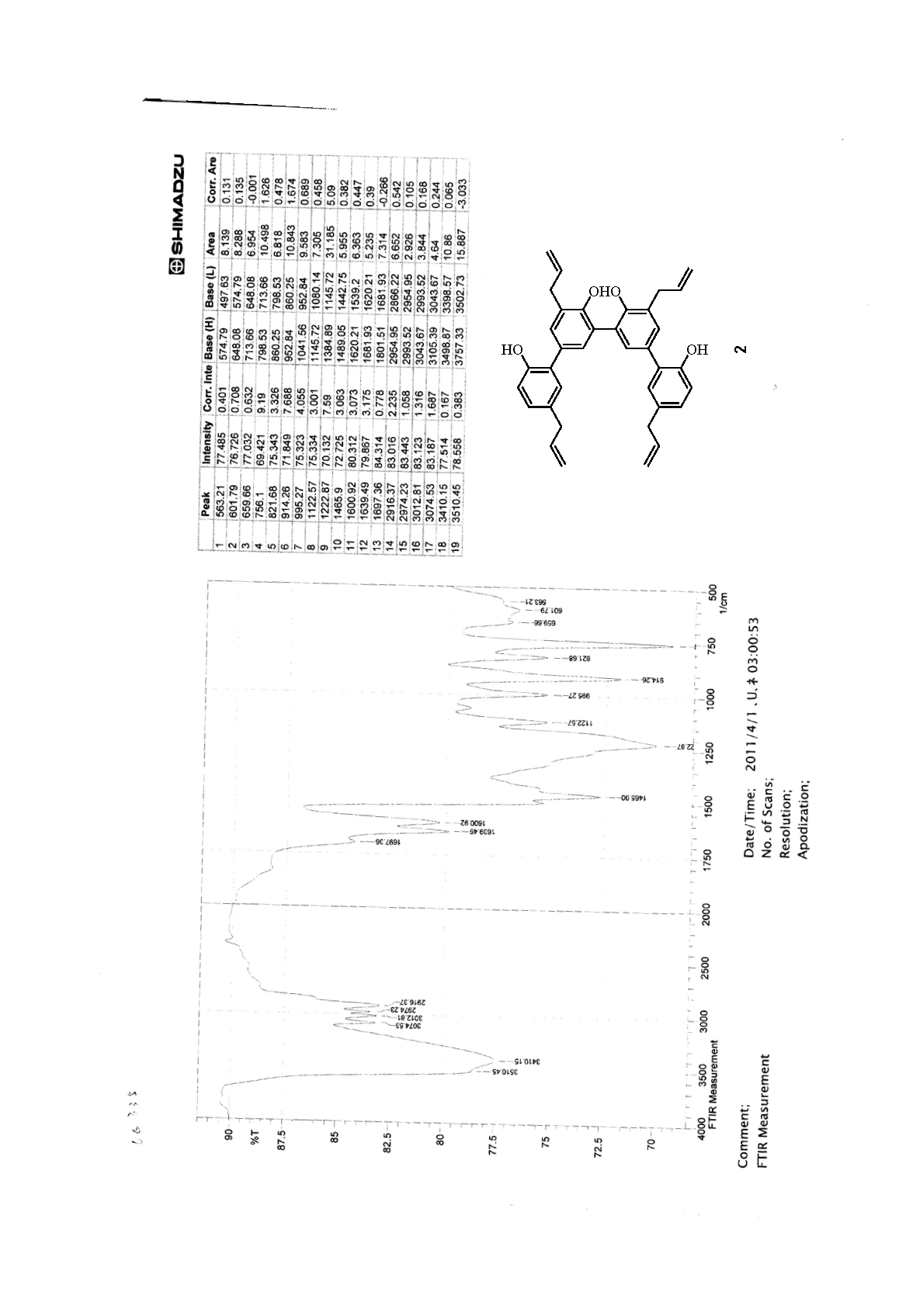

Supplement: Figure S18 — IR Spectrum of Houpulin B (2). (TIFF) [file pone.0059502.s018.tiff]

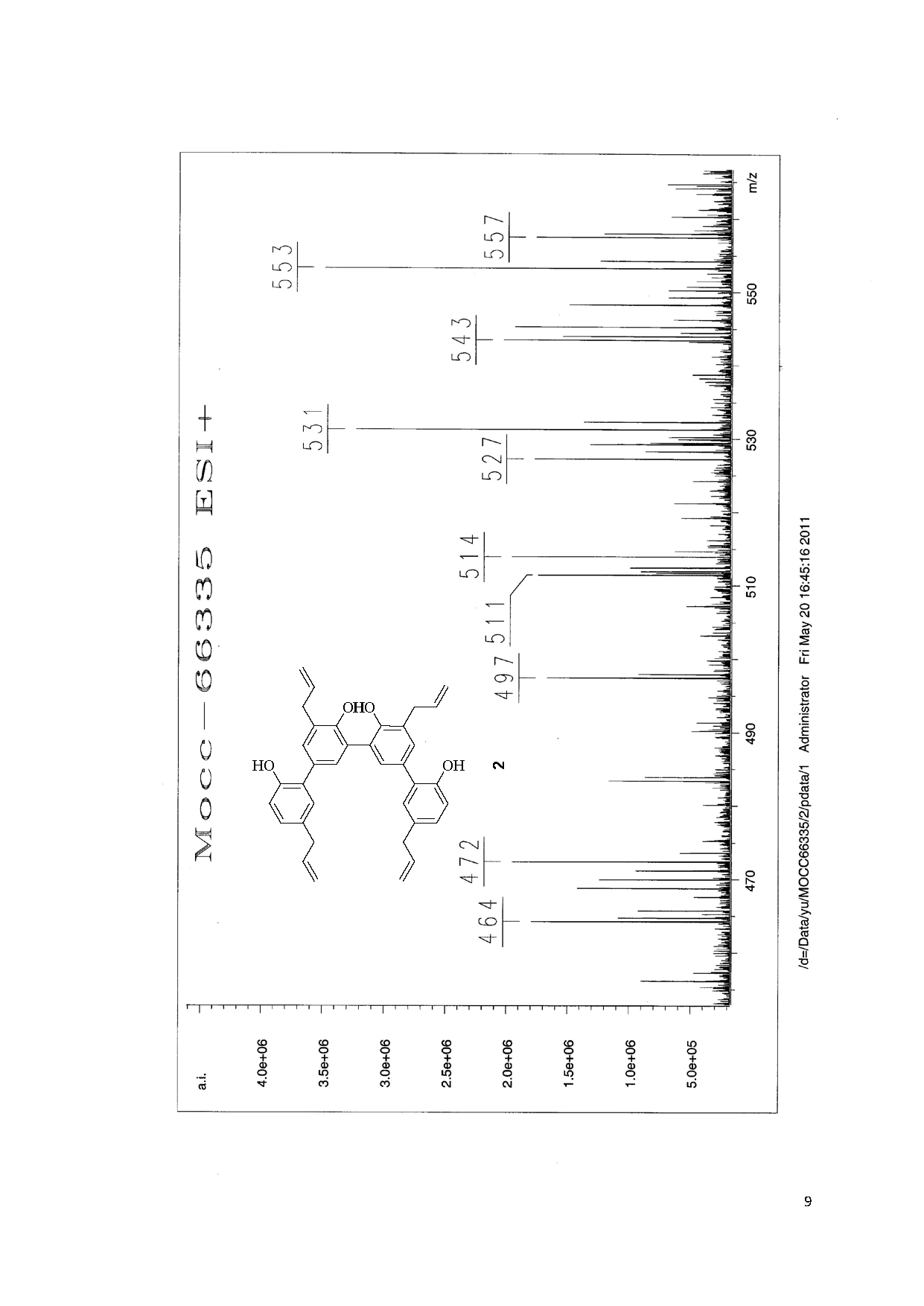

Supplement: Figure S19 — Mass Spectrum of Houpulin B (2). (TIFF) [file pone.0059502.s019.tiff]

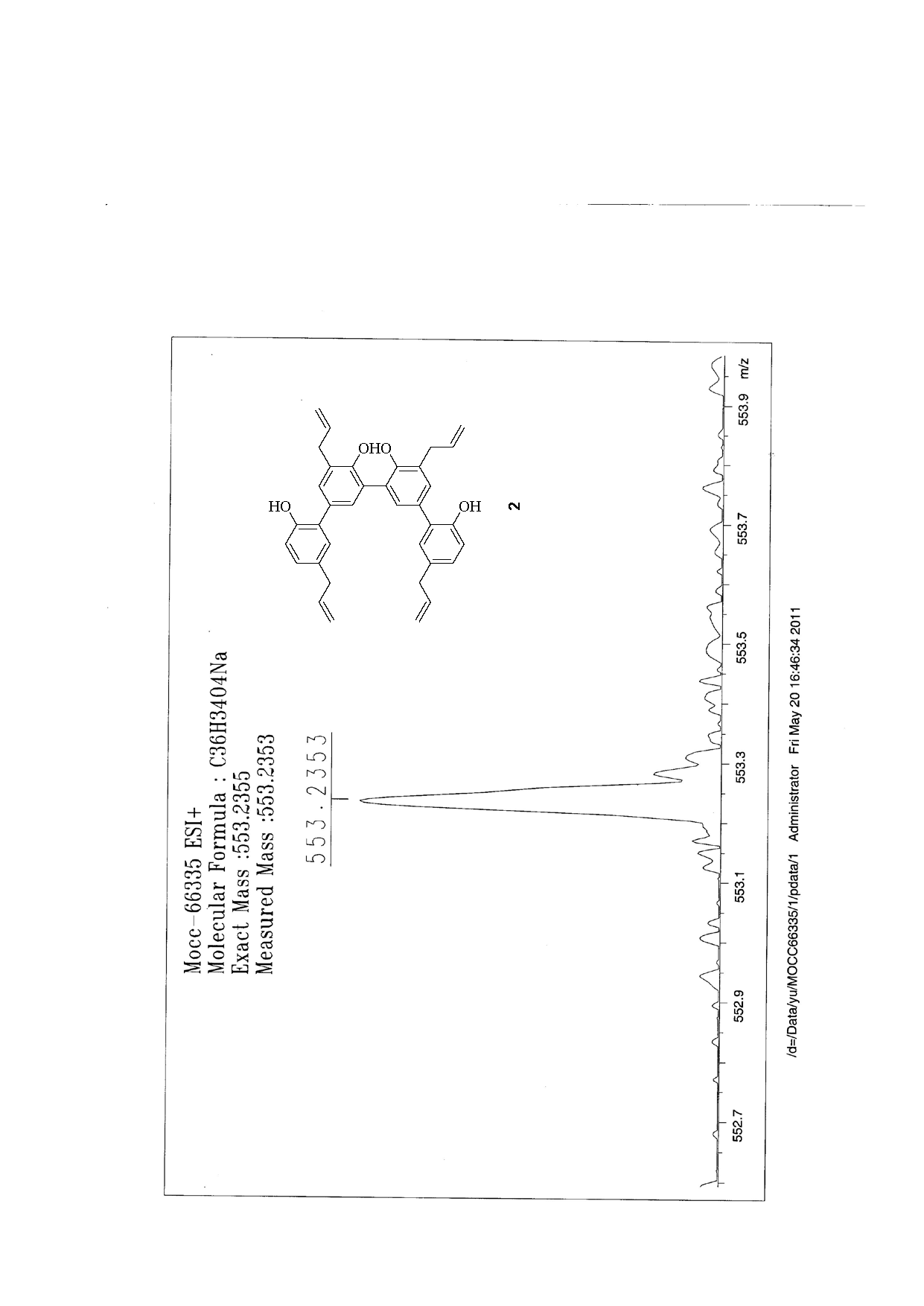

Supplement: Figure S20 — High Resolution Mass Spectrum of Houpulin B (2). (TIFF) [file pone.0059502.s020.tiff]

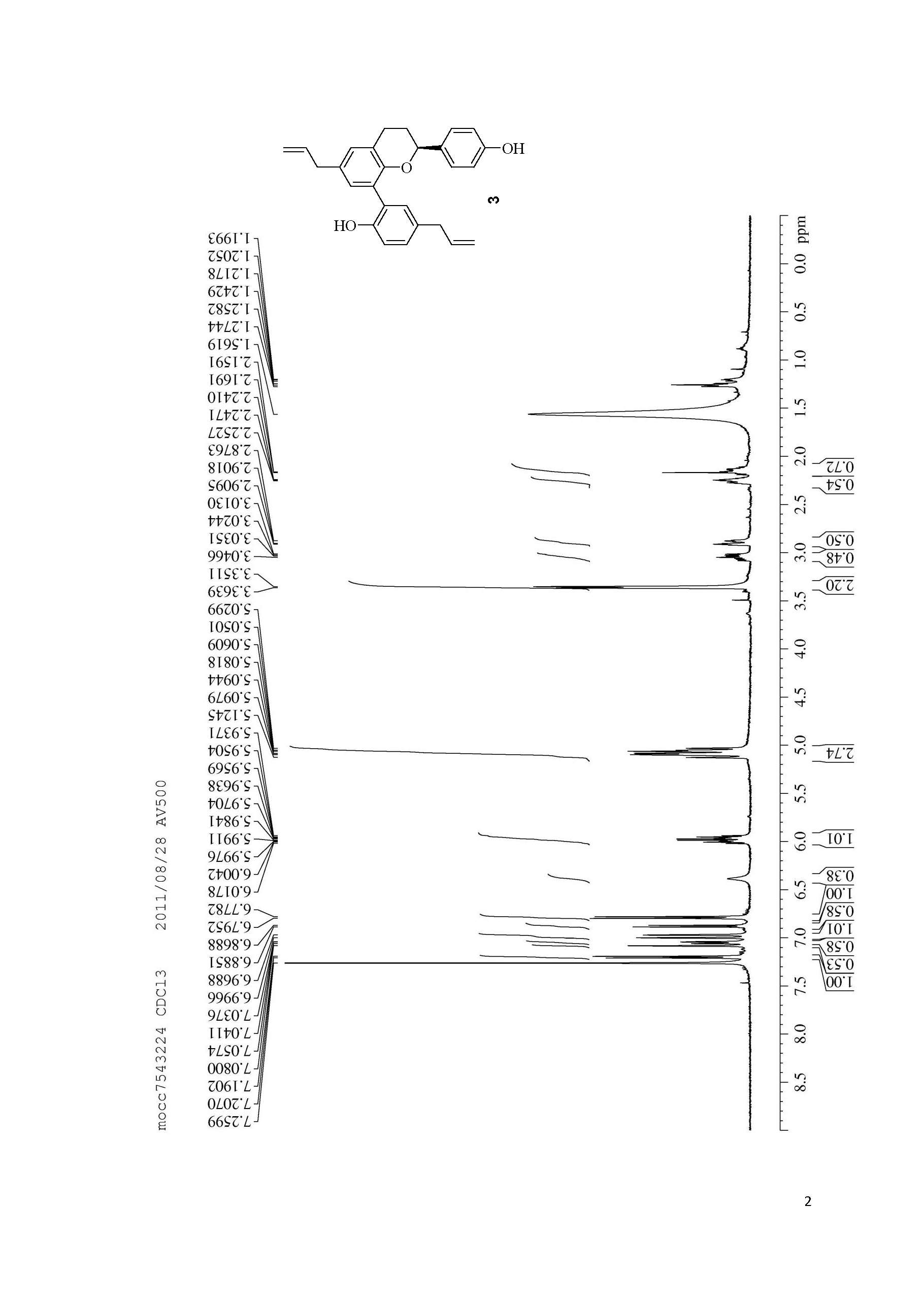

Supplement: Figure S21 — 1H NMR Spectrum of Houpulin C (3). (TIFF) [file pone.0059502.s021.tiff]

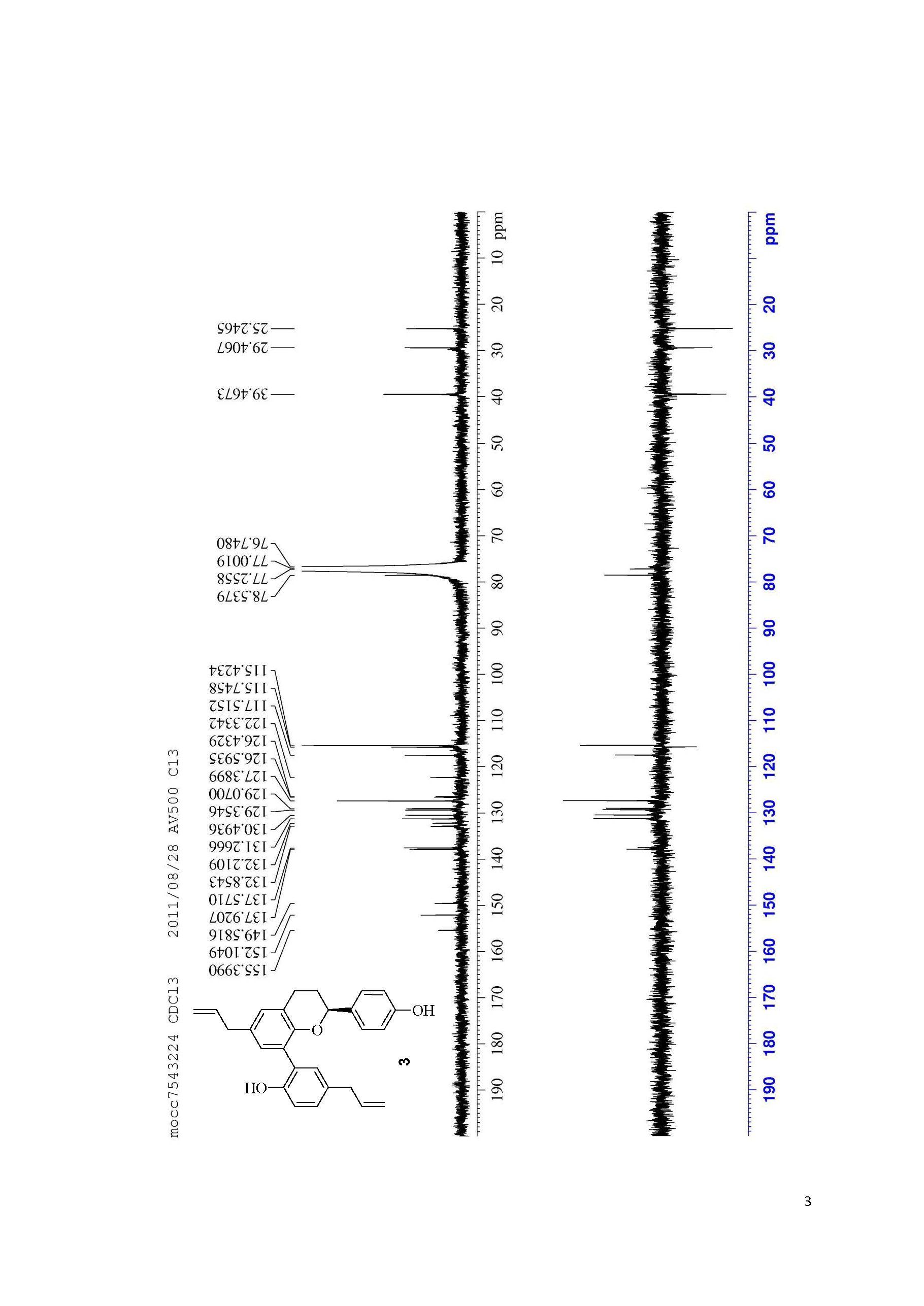

Supplement: Figure S22 — 13C and DEPT135 Spectra of Houpulin C (3). (TIFF) [file pone.0059502.s022.tiff]

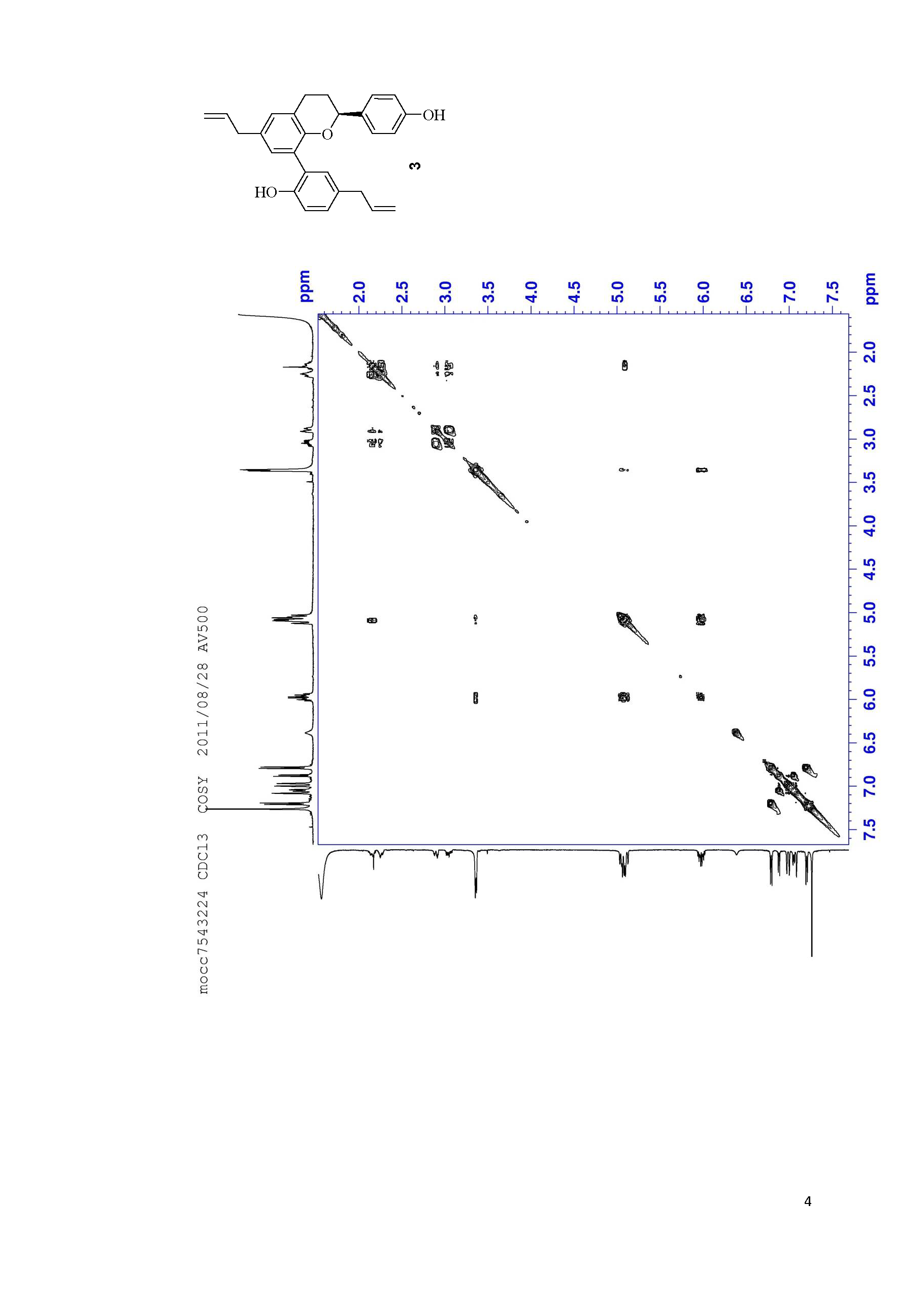

Supplement: Figure S23 — COSY Spectrum of Houpulin C (3). (TIFF) [file pone.0059502.s023.tiff]

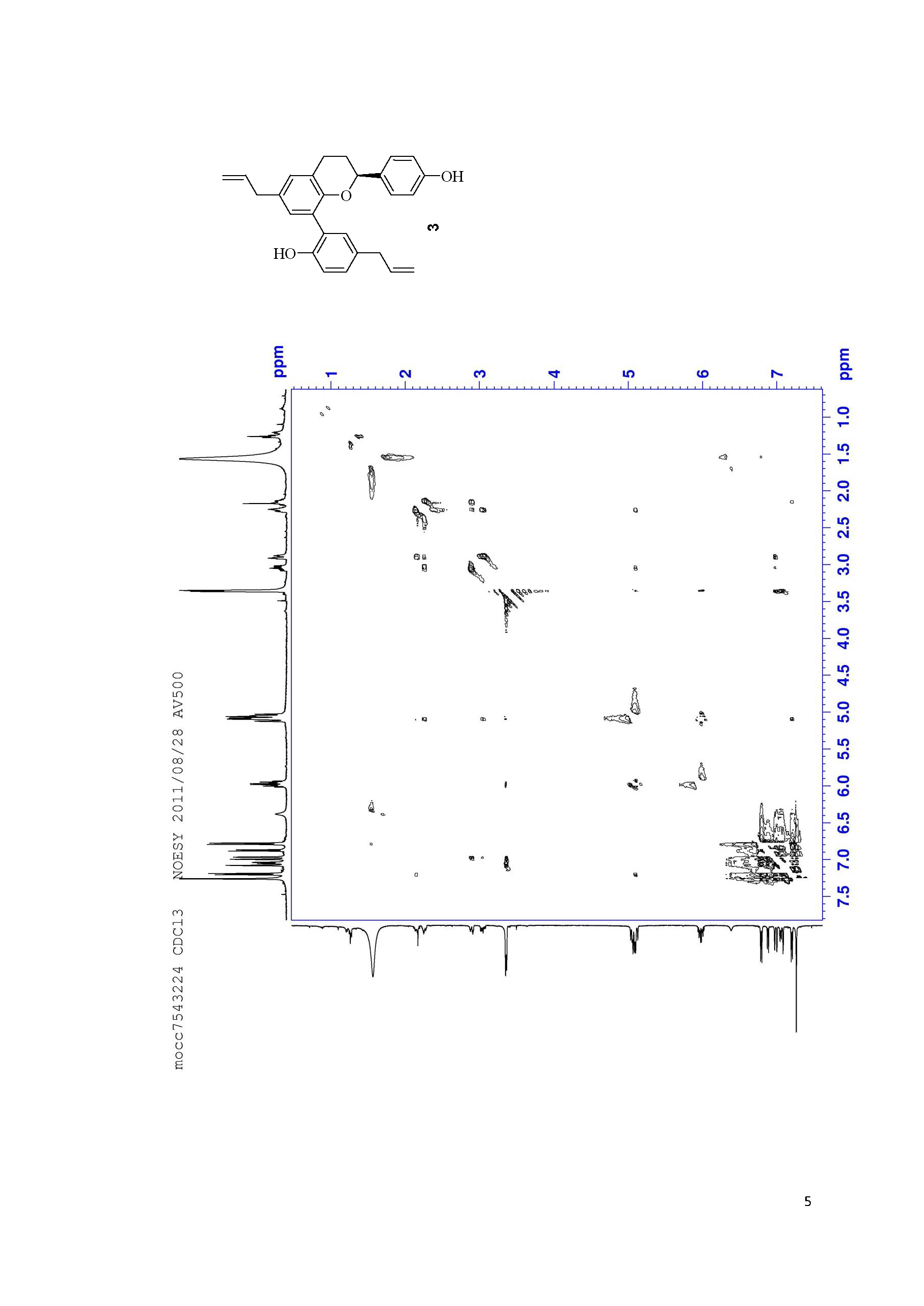

Supplement: Figure S24 — NOESY Spectrum of Houpulin C (3). (TIFF) [file pone.0059502.s024.tiff]

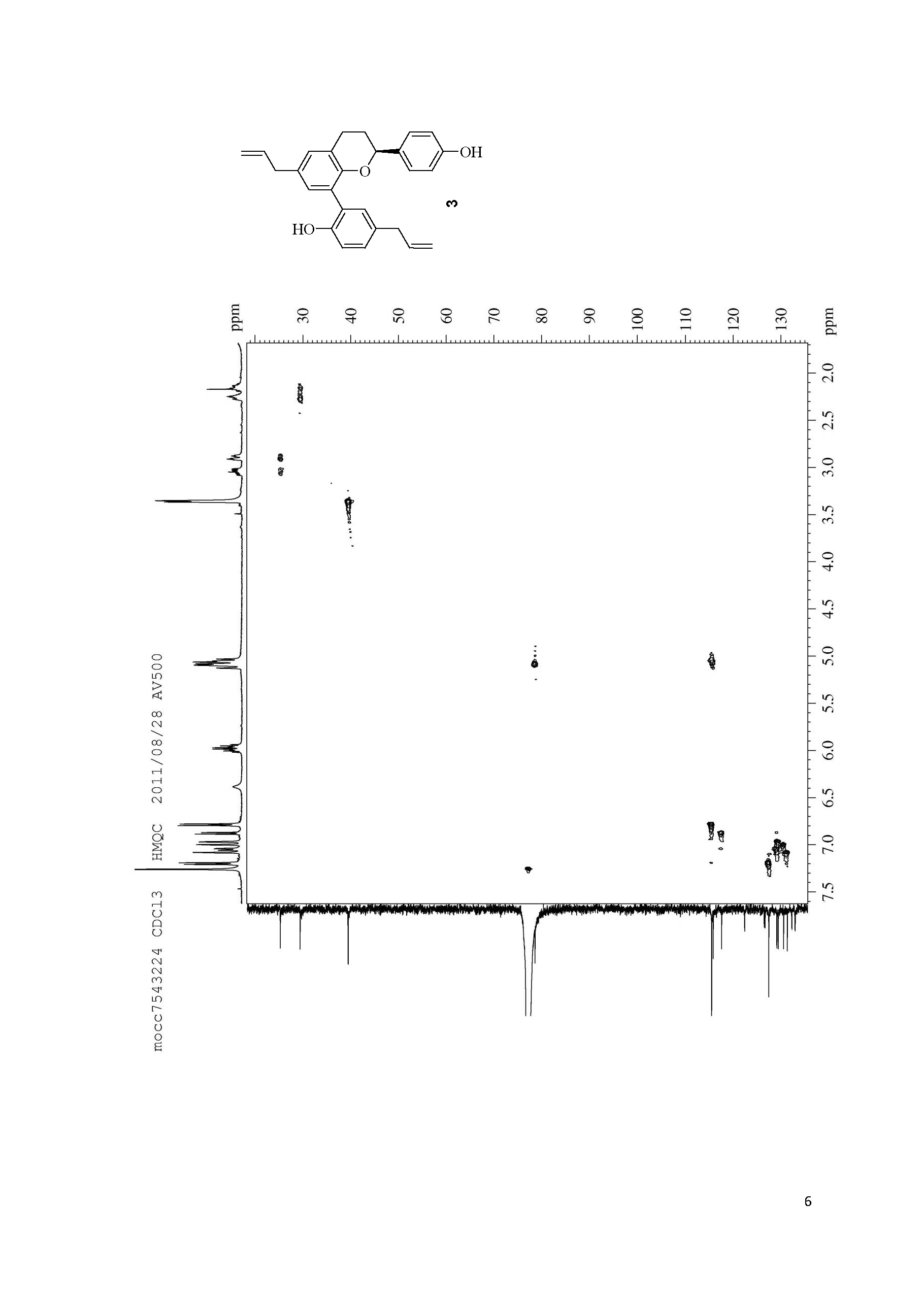

Supplement: Figure S25 — HMQC Spectrum of Houpulin C (3). (TIFF) [file pone.0059502.s025.tiff]

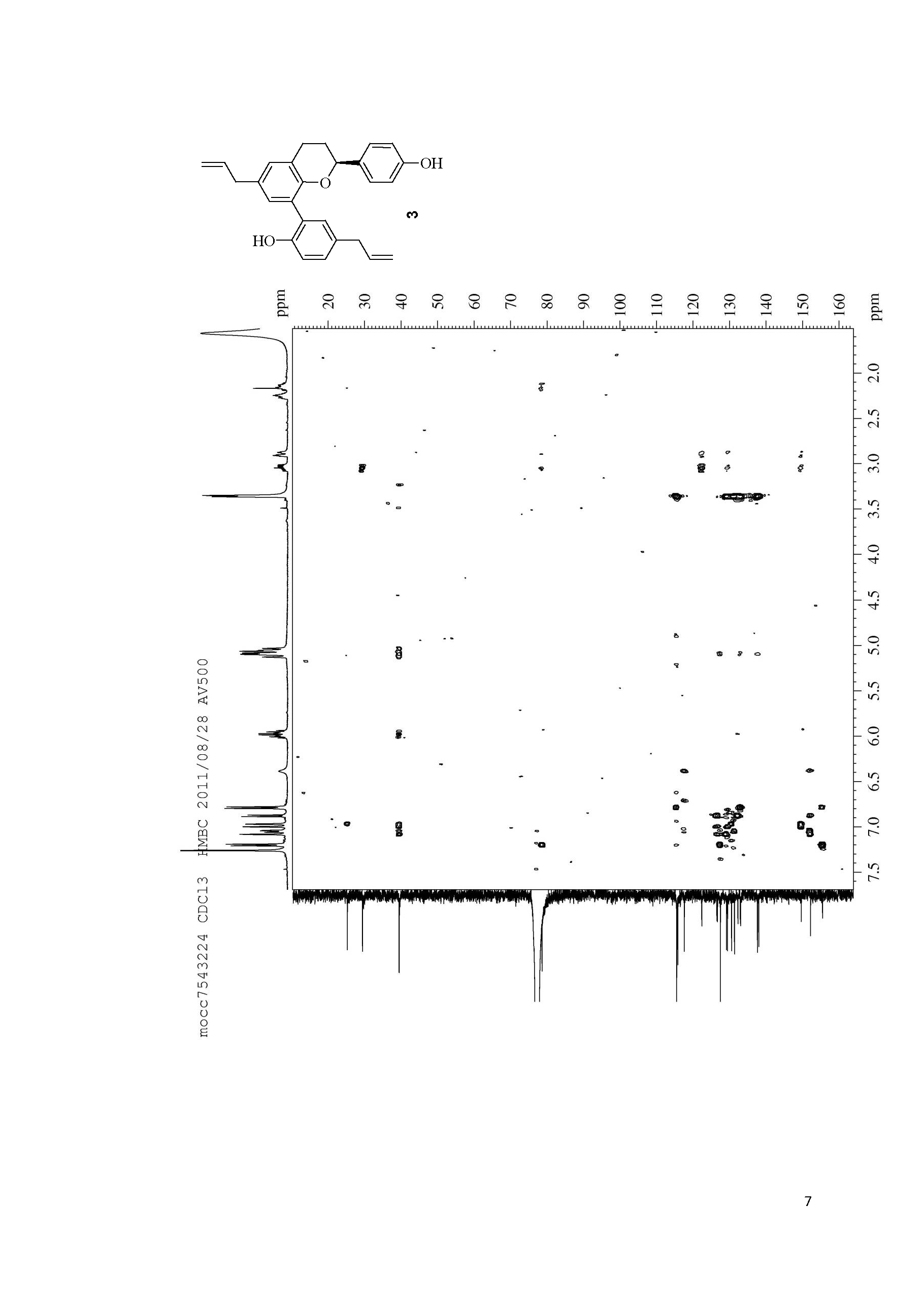

Supplement: Figure S26 — HMBC Spectrum of Houpulin C (3). (TIFF) [file pone.0059502.s026.tiff]

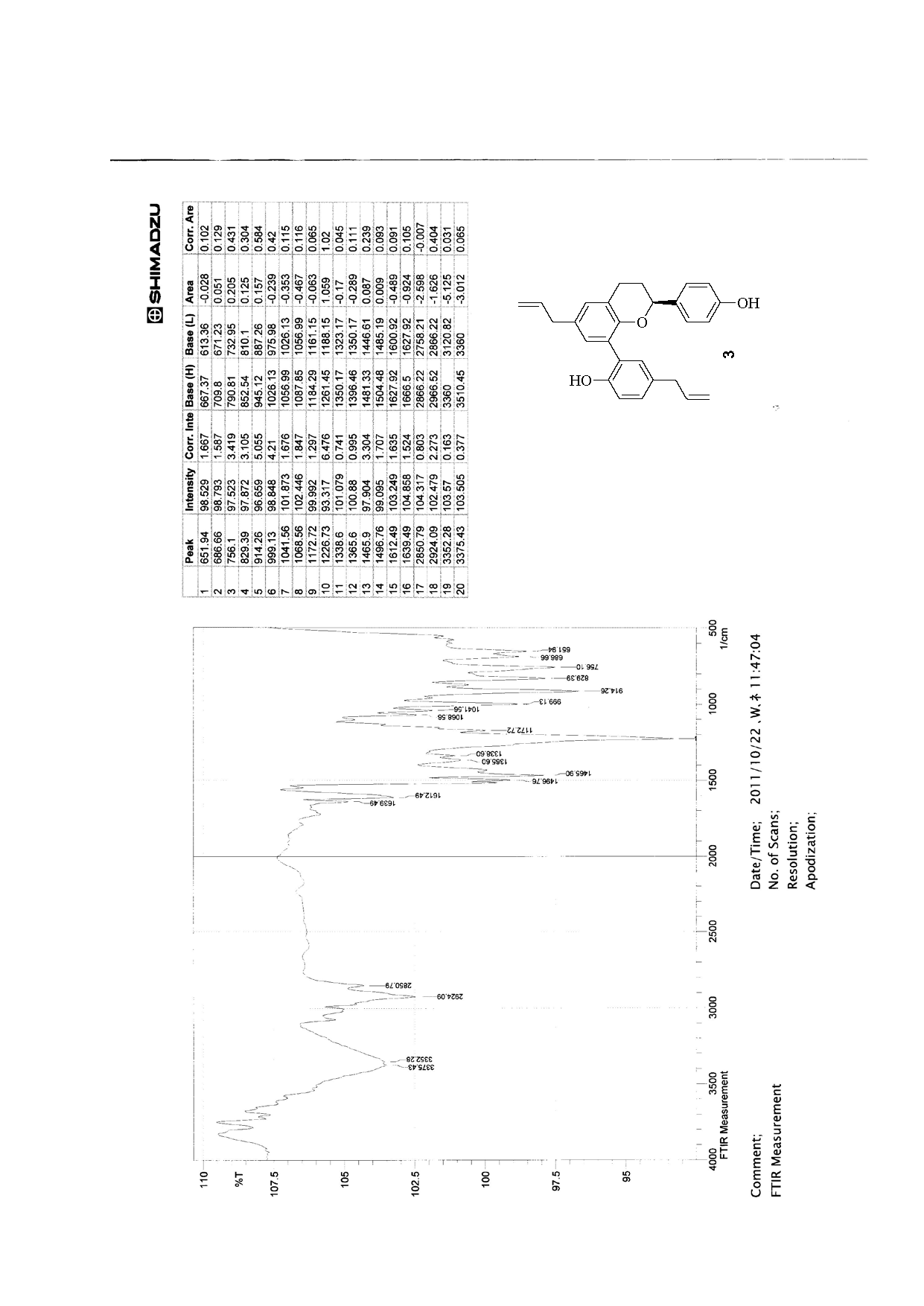

Supplement: Figure S27 — IR Spectrum of Houpulin C (3). (TIFF) [file pone.0059502.s027.tiff]

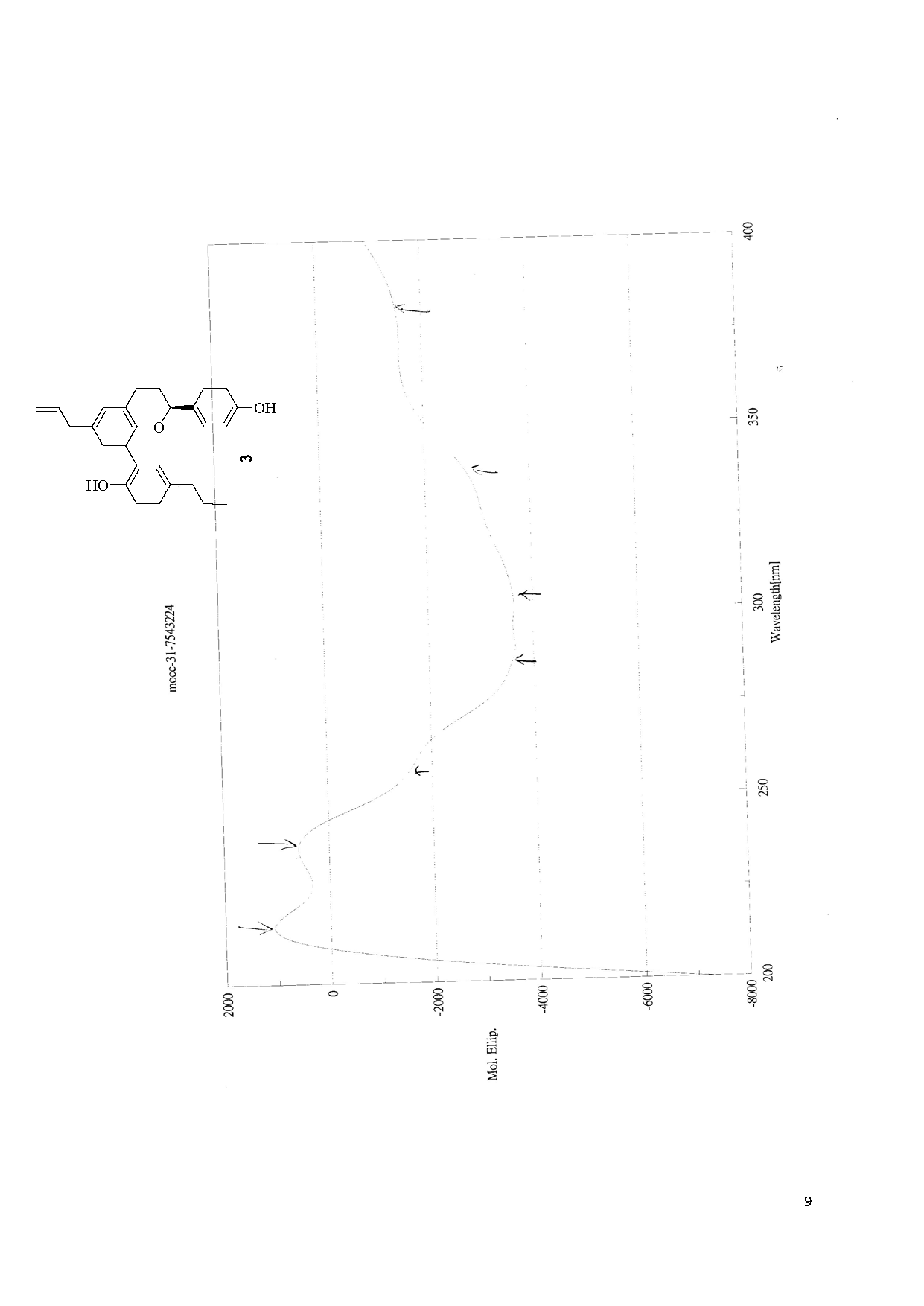

Supplement: Figure S28 — CD Spectrum of Houpulin C (3). (TIFF) [file pone.0059502.s028.tiff]

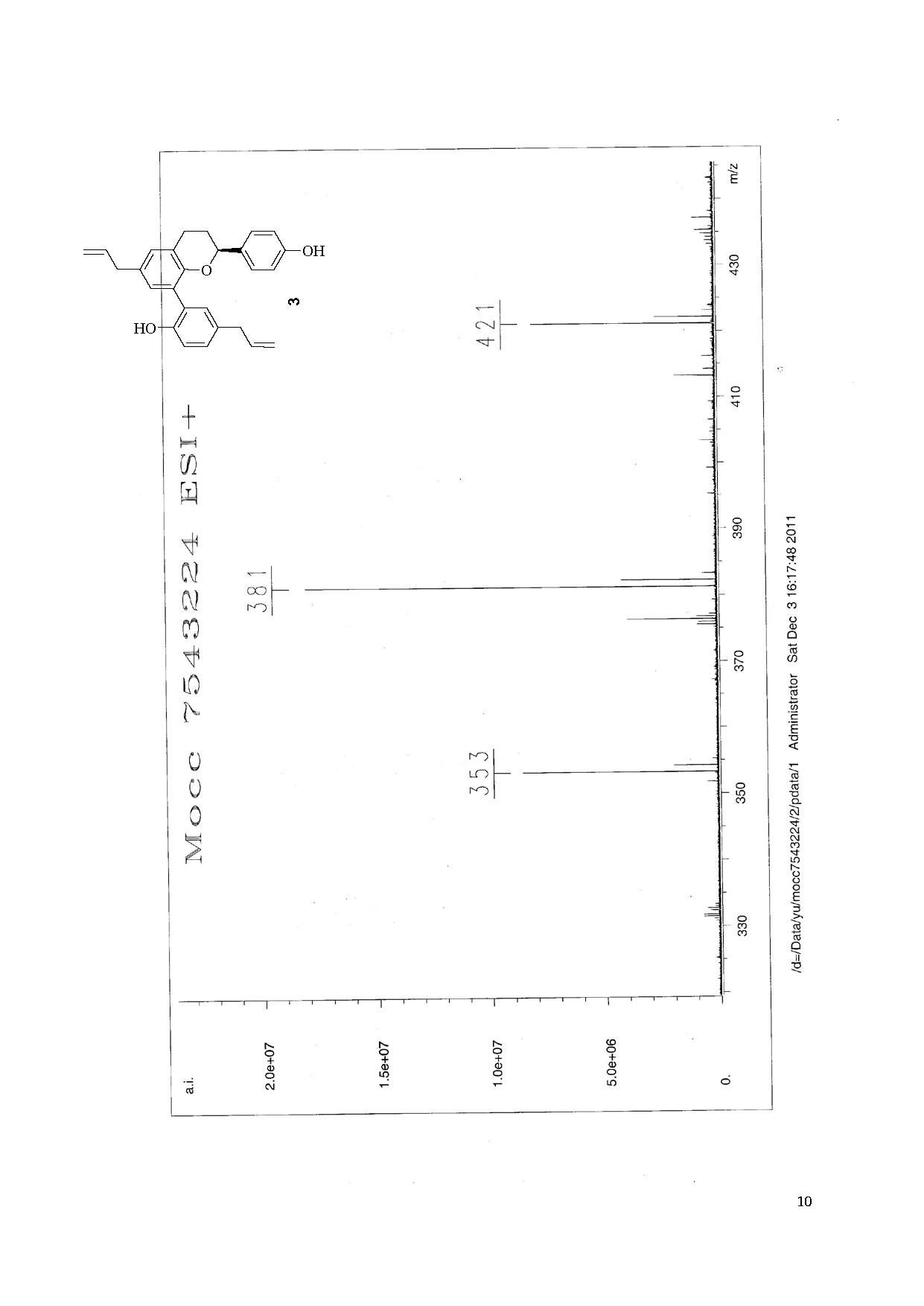

Supplement: Figure S29 — Mass Spectrum of Houpulin C (3). (TIFF) [file pone.0059502.s029.tiff]

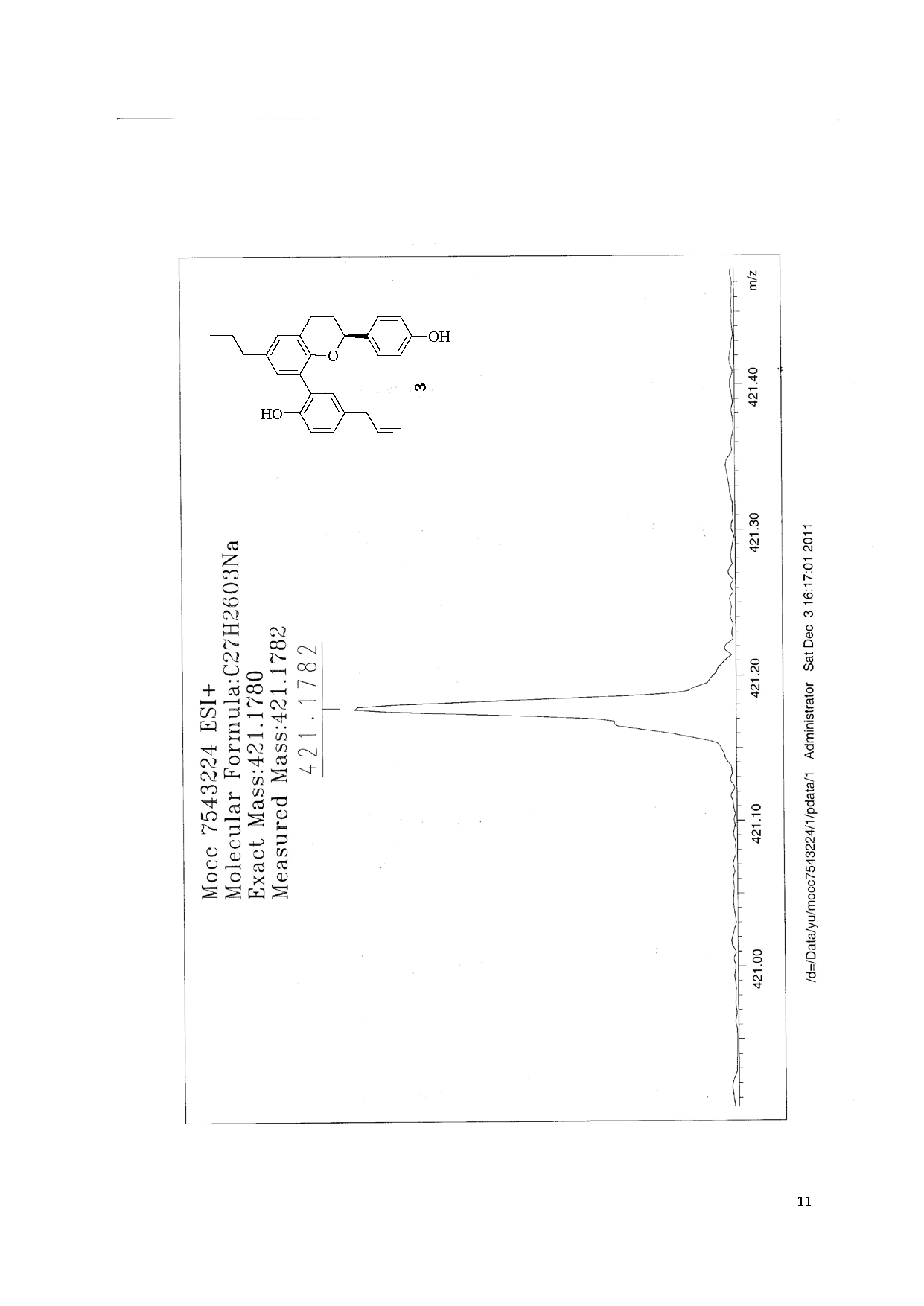

Supplement: Figure S30 — High Resolution Mass Spectrum of Houpulin C (3). (TIFF) [file pone.0059502.s030.tiff]

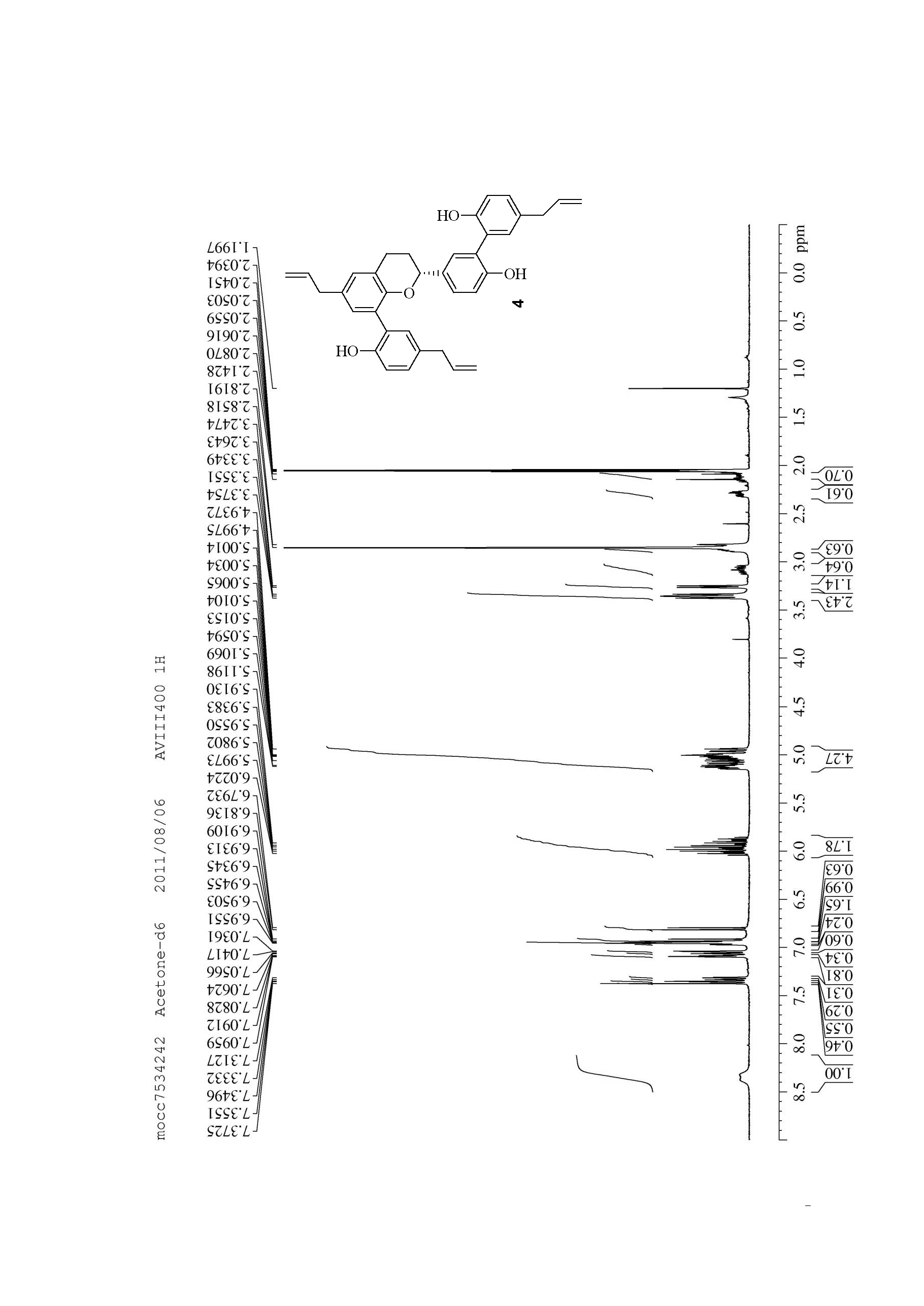

Supplement: Figure S31 — 1H NMR Spectrum of Houpulin D (4). (TIFF) [file pone.0059502.s031.tiff]

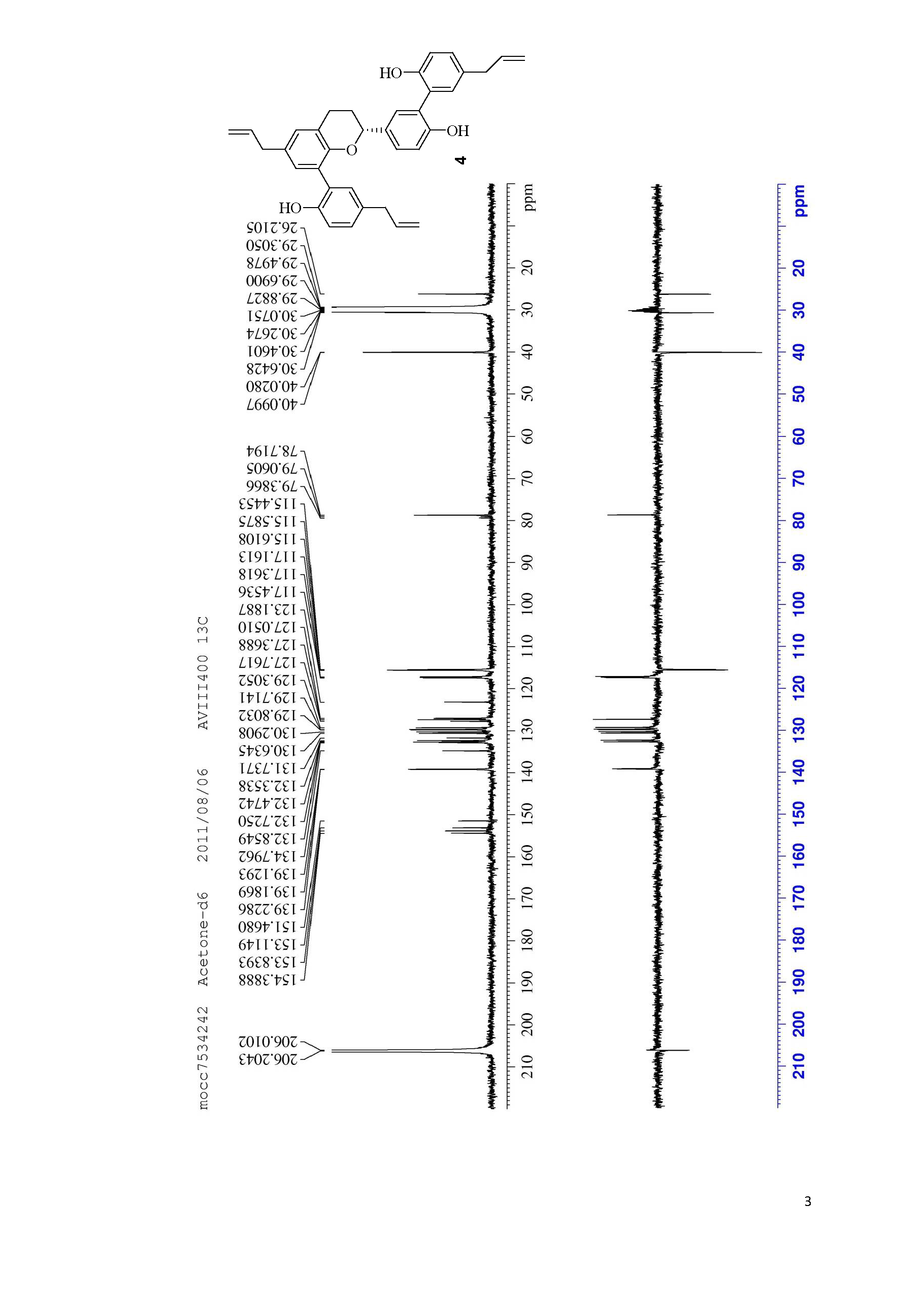

Supplement: Figure S32 — 13C and DEPT135 Spectra of Houpulin D (4). (TIFF) [file pone.0059502.s032.tiff]

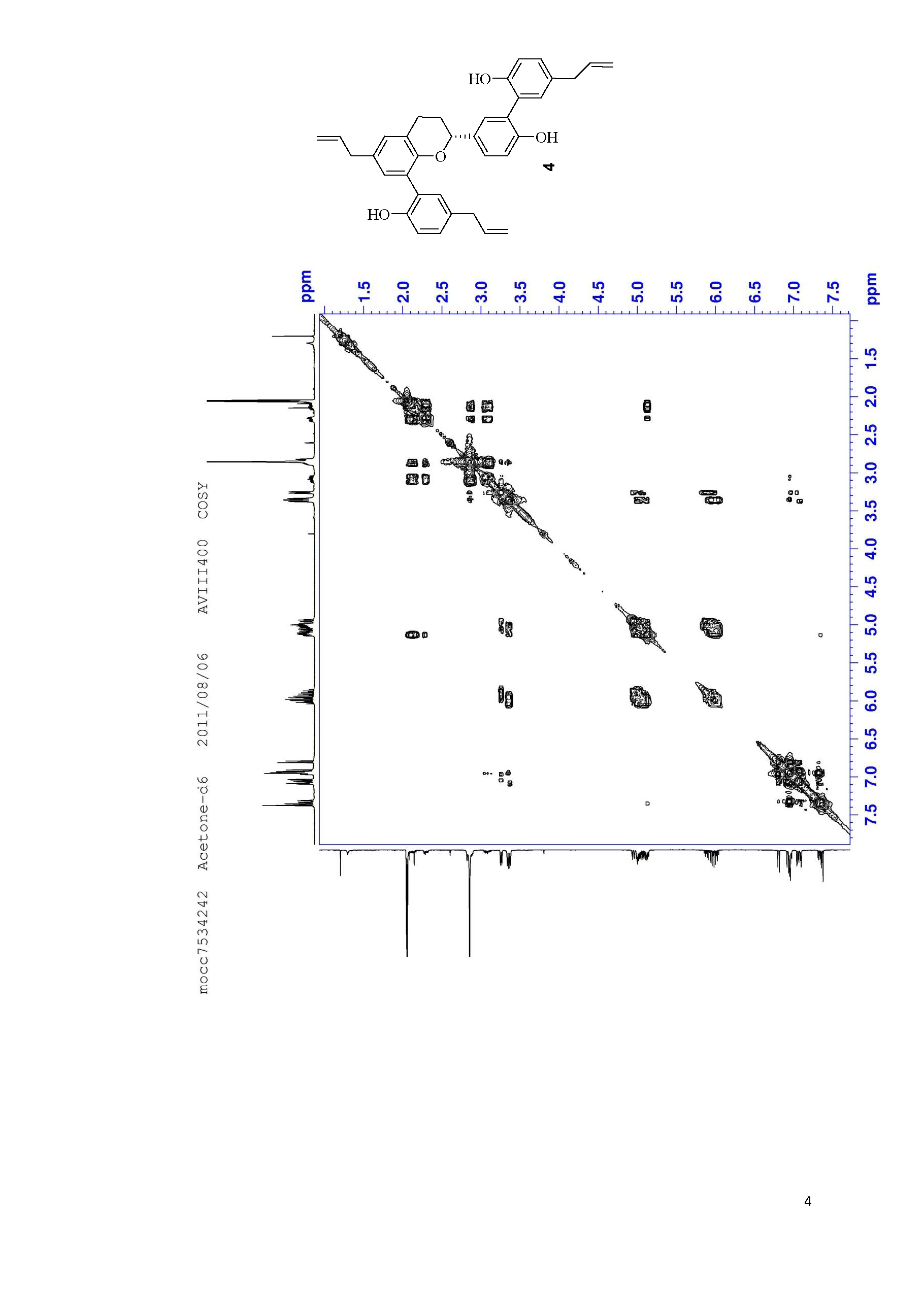

Supplement: Figure S33 — COSY Spectrum of Houpulin D (4). (TIFF) [file pone.0059502.s033.tiff]

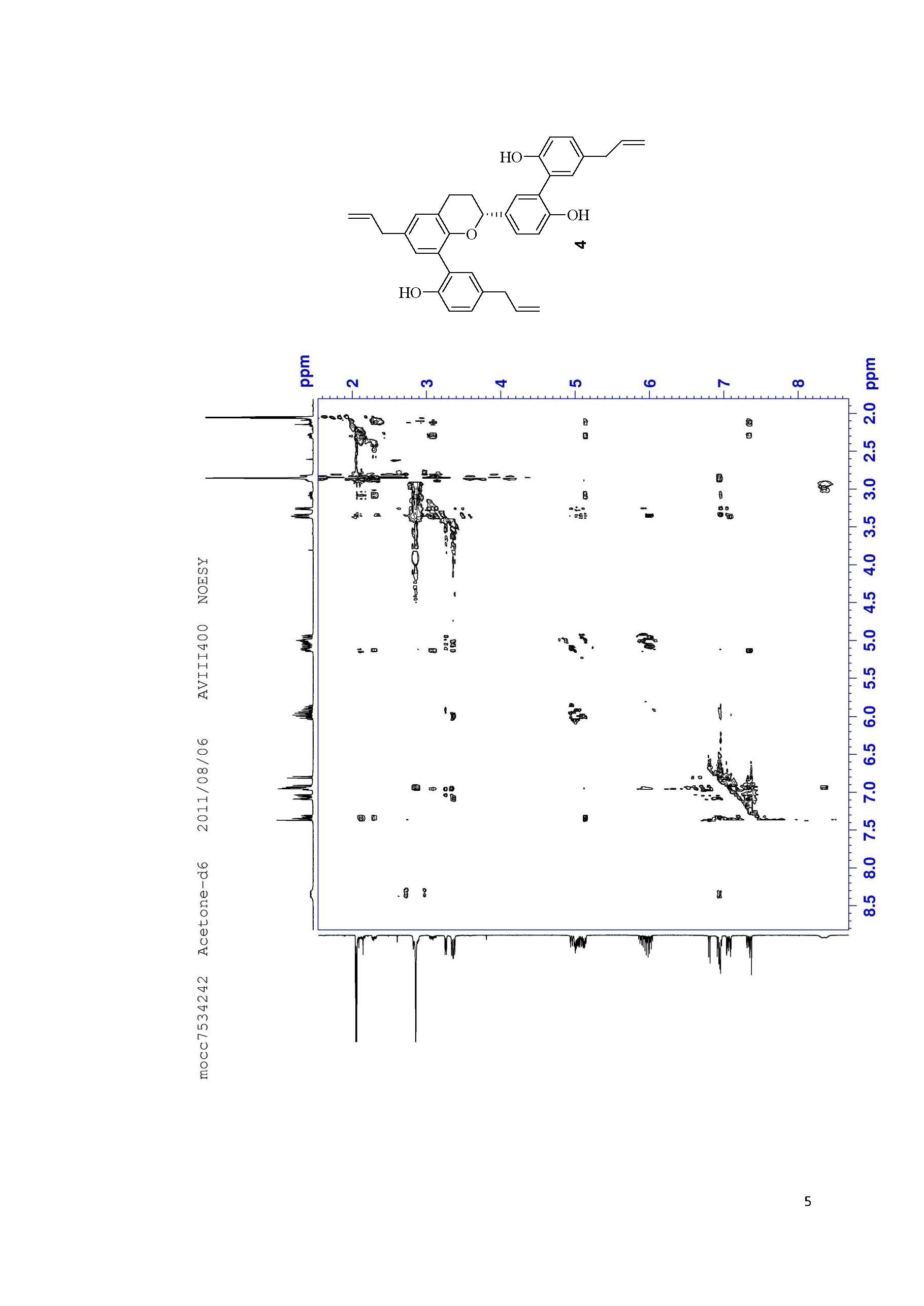

Supplement: Figure S34 — NOESY Spectrum of Houpulin D (4). (TIFF) [file pone.0059502.s034.tiff]

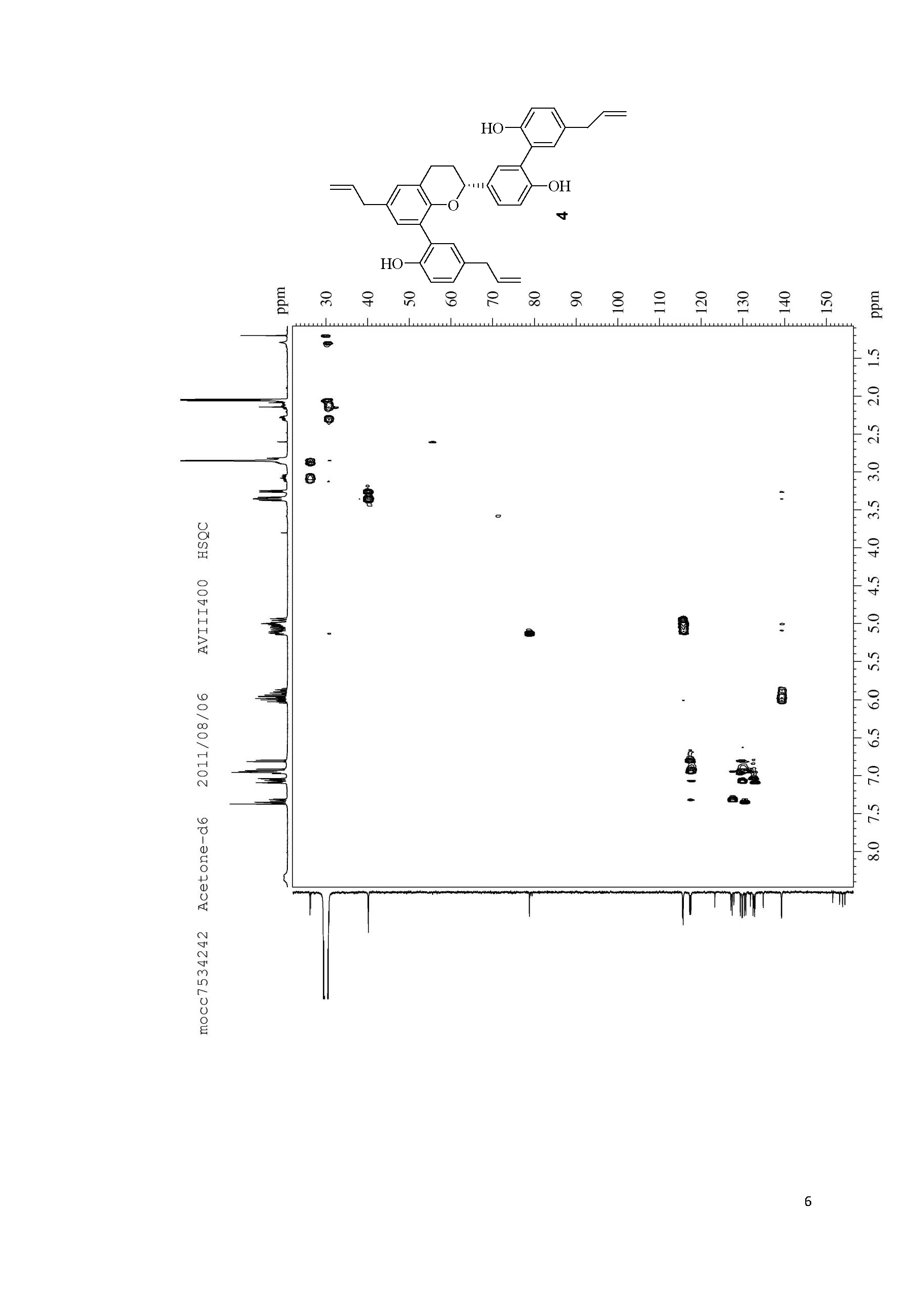

Supplement: Figure S35 — HSQC Spectrum of Houpulin D (4). (TIFF) [file pone.0059502.s035.tiff]

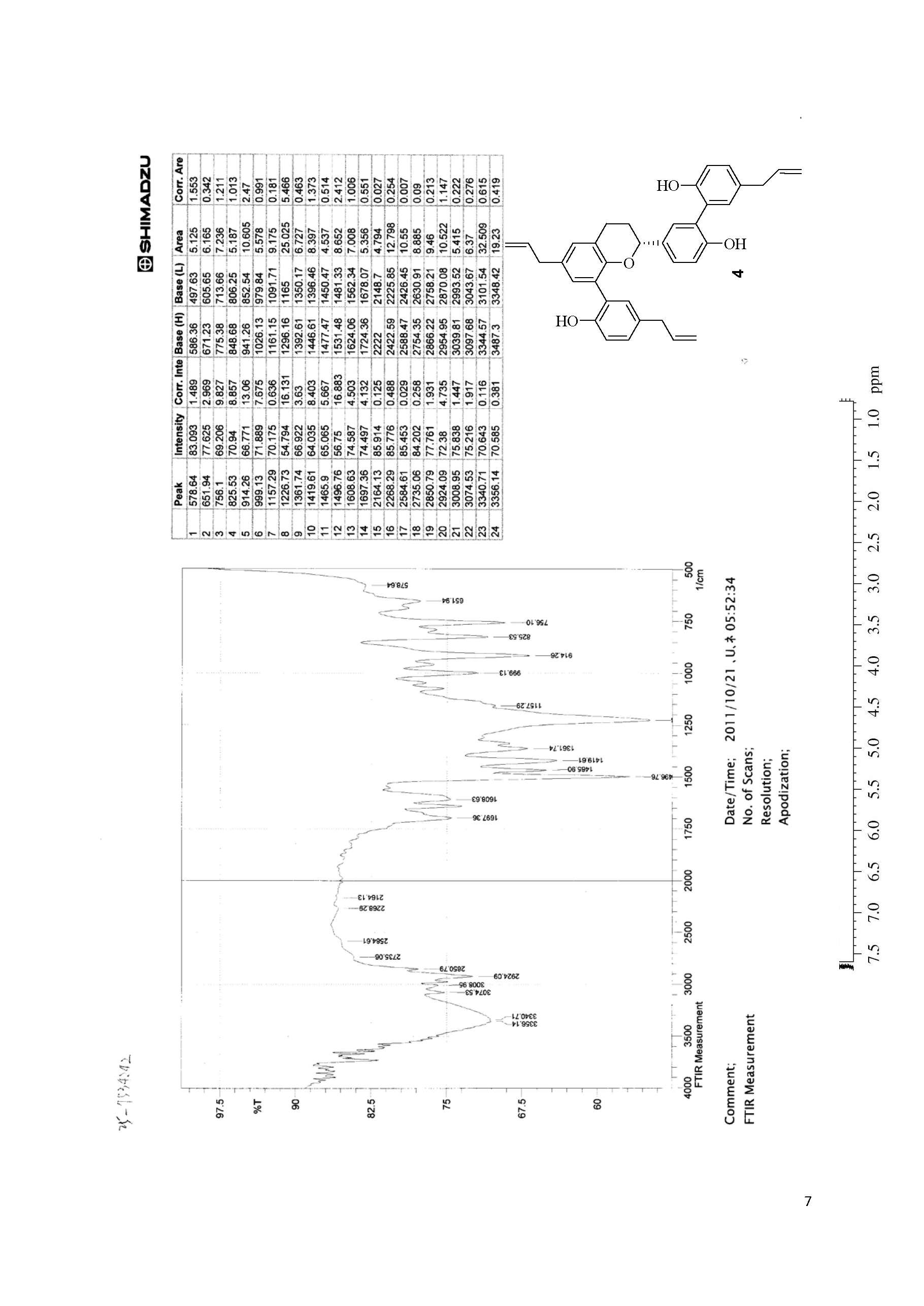

Supplement: Figure S36 — IR Spectrum of Houpulin D (4). (TIFF) [file pone.0059502.s036.tiff]

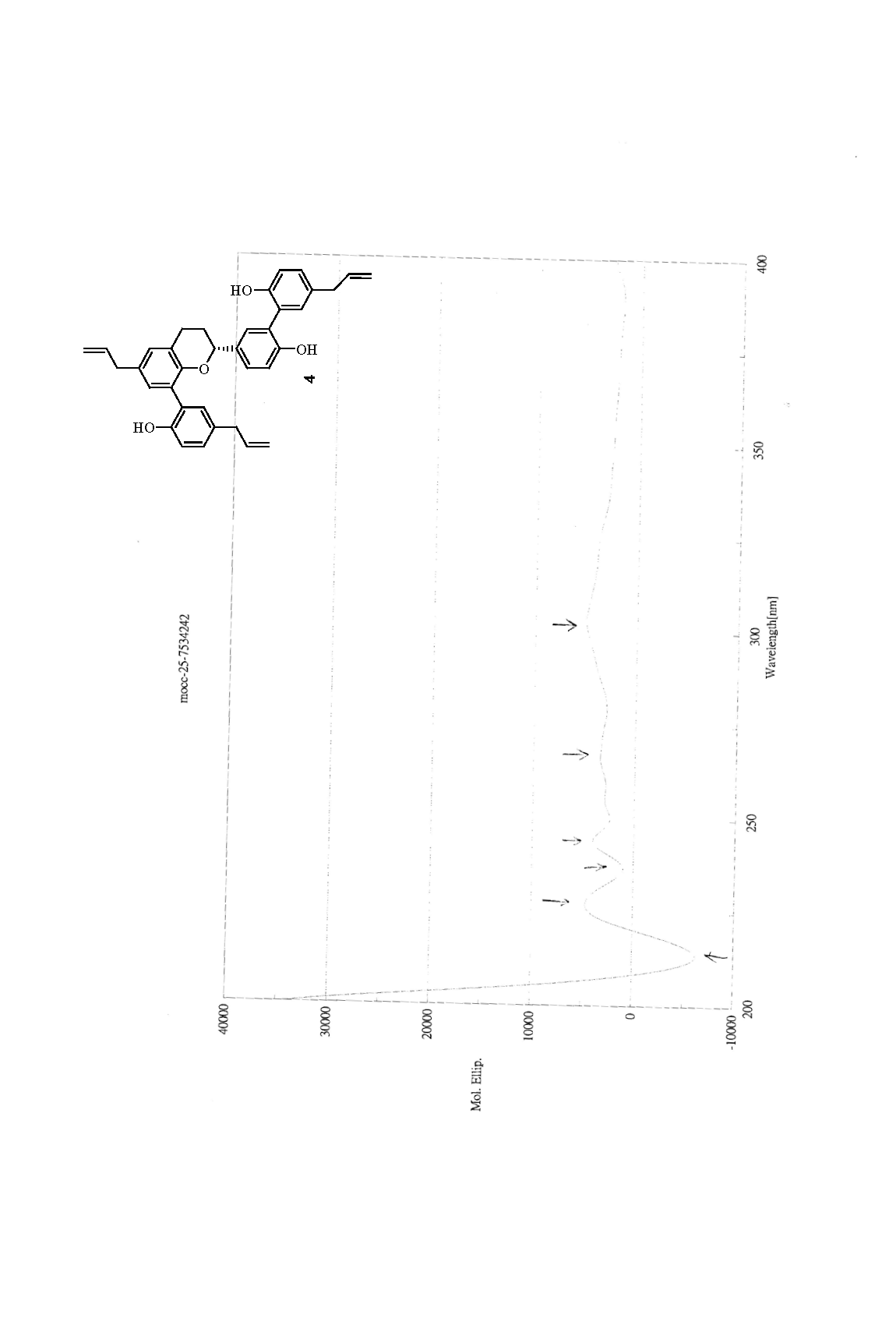

Supplement: Figure S37 — CD Spectrum of Houpulin D (4). (TIFF) [file pone.0059502.s037.tiff]

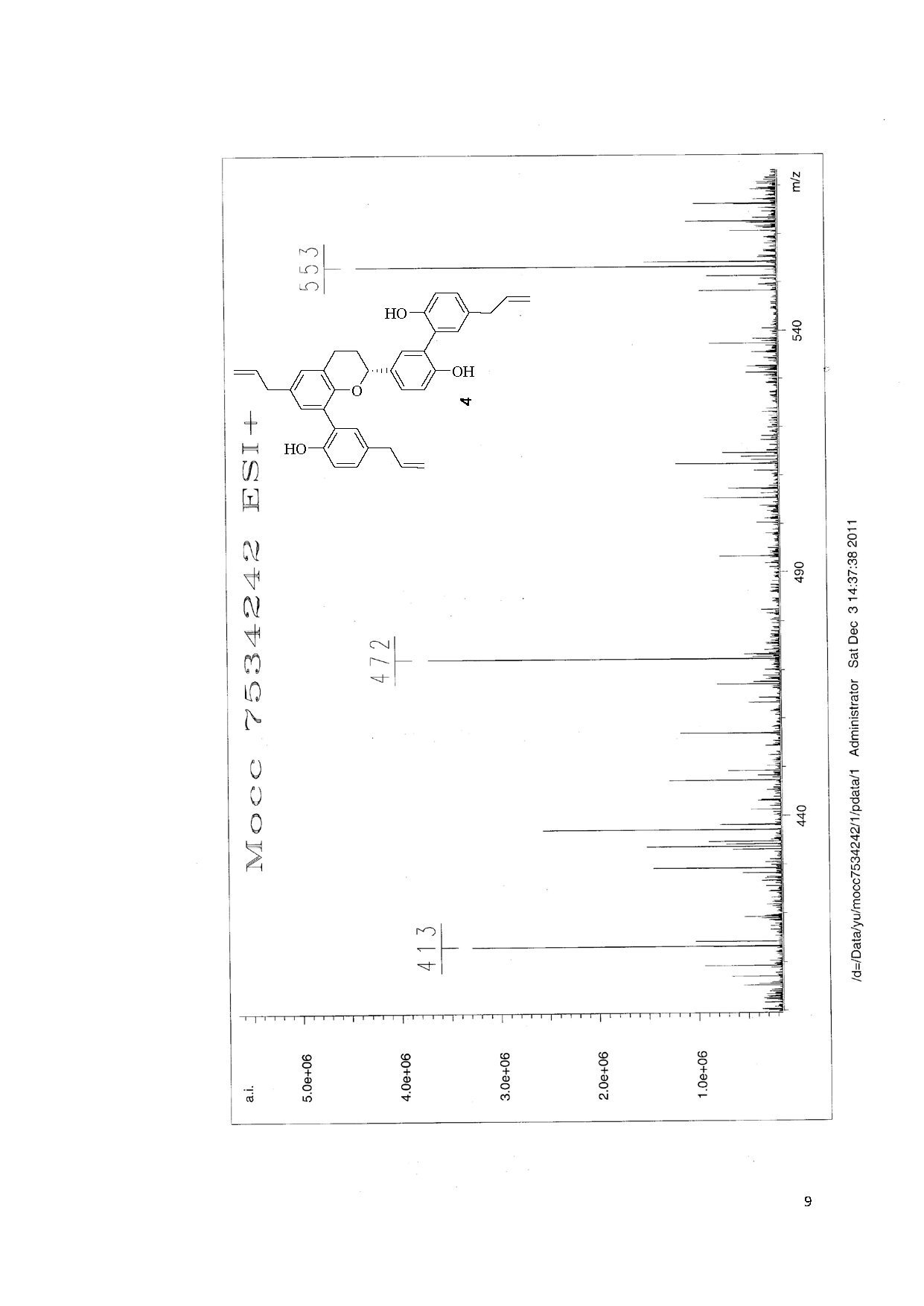

Supplement: Figure S38 — Mass Spectrum of Houpulin D (4). (TIFF) [file pone.0059502.s038.tiff]

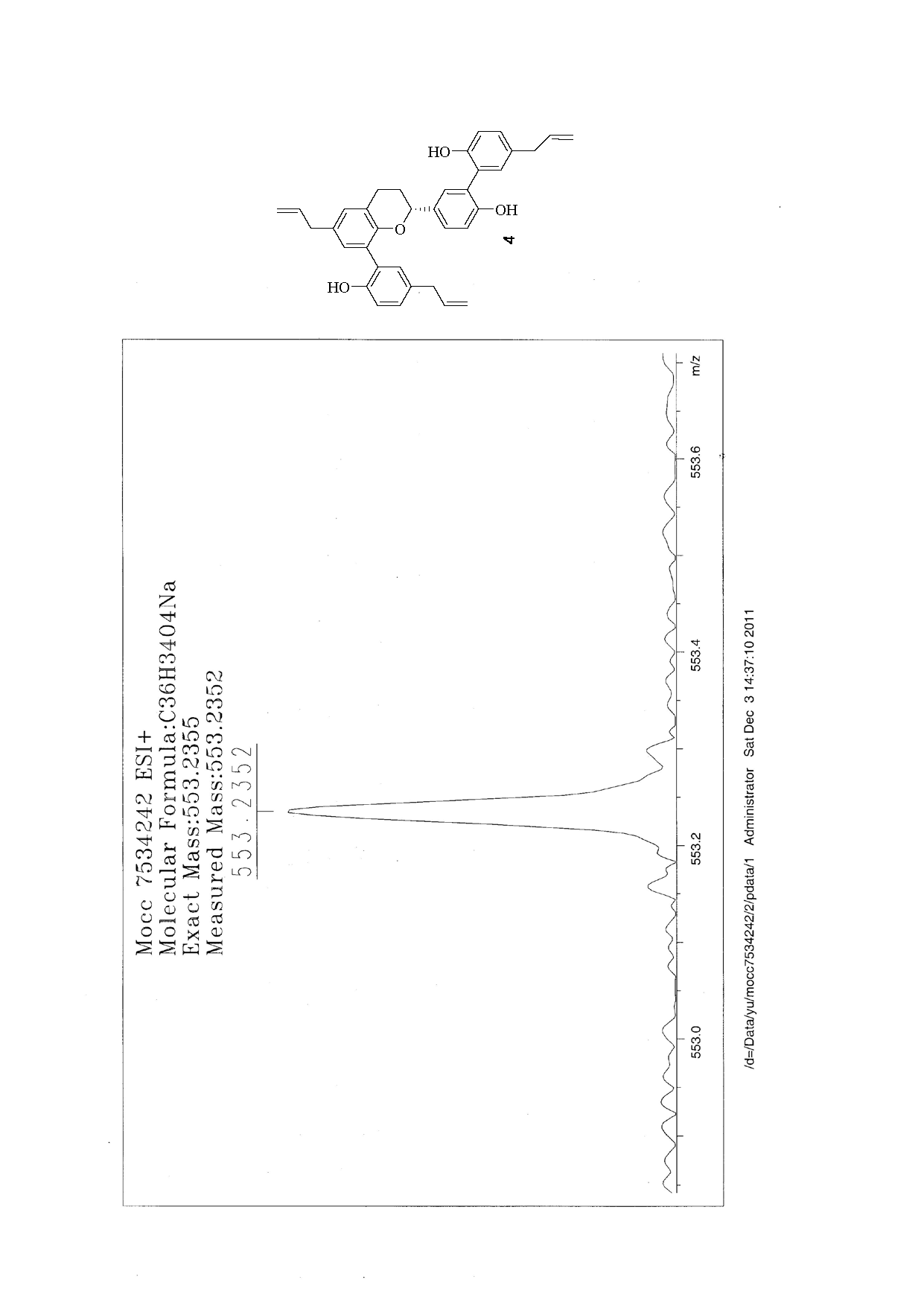

Supplement: Figure S39 — High Resolution Mass Spectrum of Houpulin D (4). (TIFF) [file pone.0059502.s039.tiff]
